# Reducing Obesity Using Social Ties (ROBUST): Protocol for a randomized control trial of a social network lifestyle intervention

**DOI:** 10.1371/journal.pone.0318990

**Published:** 2025-04-16

**Authors:** Erica Phillips, Caitlin Potter, Jaleel Poole, Anika Lewis, Mussarat Nahid, Paul Christos, Katie Hootman, Ginger Winston, Kayla de la Haye

**Affiliations:** 1 Department of Medicine, Division of General Internal Medicine, Weill Cornell Medicine, New York, New York, United States of America; 2 Department of Nutritional Sciences, College of Human Ecology, Cornell University, Ithaca, New York, United States of America; 3 Department of Population Health Sciences, Division of Biostatistics, Weill Cornell Medicine, New York, New York, United States of America; 4 Clinical and Translational Science Center, Weill Cornell Medicine, New York, New York, United States of America; 5 Food and Drug Administration, Office of Cardiology, Hematology, Endocrinology and Nephrology, Center for the Drug Evaluation and Research, Silver Spring, Maryland, United States of America; 6 Institute for Food System Equity, Center for Economic and Social Research, Dornsife College of Letters, Arts and Sciences, University of Southern California, Los Angeles, California, United States of America; PLOS: Public Library of Science, UNITED KINGDOM OF GREAT BRITAIN AND NORTHERN IRELAND

## Abstract

**Aim:**

The aim of the Reducing Obesity Using Social Ties (ROBUST) study is to address negative and positive social network processes that may influence weight loss among individuals enrolled in an evidence-based, 24-week lifestyle intervention modeled after the Diabetes Prevention Program.

**Methods:**

This is a randomized controlled trial (RCT) of a tailored social network-enhanced lifestyle intervention compared to an individual-level lifestyle intervention (control group) in adults (N=132) who self-identify as Black or Hispanic and have obesity (BMI ≥ 30 kg/m^2^). Intervention participants will recruit up to two members (n=132) of their social network to participate in skill-building sessions. Intervention and control participants will receive fourteen nutrition and lifestyle coaching sessions over 24 weeks. Diet, physical activity, positive affect, social network processes, communal coping, and anthropometrics will be measured at baseline and after intervention completion at 24 weeks.

**Discussion:**

This study will provide the first pragmatic evidence of the efficacy of activating a communal coping process within personal networks to optimize weight loss among Black and Hispanic adults. Our study explicitly targets a population that has been less responsive to other lifestyle approaches to weight loss.

**Trial registration:**

The study protocol was prospectively registered on ClinicalTrials.gov (NCT06335810).

## Introduction

Eliminating the disproportionate burden of obesity and obesity-associated chronic health conditions (i.e., Type 2 Diabetes, hypertension) experienced by adults who self-identify as non-Hispanic Black or Hispanic remains a pervasive challenge for the United States healthcare system. Non-Hispanic Black adults have the highest prevalence of obesity (49.6%) compared with all other races (non-Hispanic Asian 17.4%; non-Hispanic White 42.2%) and Hispanic-origin groups (44.8%) [1]. The continued rise in the prevalence of obesity cannot be explained by genetics or individual factors alone. It has become increasingly recognized that social and structural factors, from interpersonal relationships and social norms to health and economic policy, contribute to obesity risk and health inequities [2]. However, most state-of-the-art lifestyle and obesity interventions focus on changing individual factors and behaviors and do not include components that address the social and environmental context.

Presently, most behavioral lifestyle intervention studies result in clinically meaningful weight loss (5% or greater) amongst half of the study participants [3]. There remain notable differences in treatment response to the same intervention across different racial, ethnic, and sex groups. One such example is the Diabetes Prevention Program which demonstrated a weight-loss differential between white men (-8.4%), white women (-8.1%), Hispanic men (-7.8%), Hispanic women (-7.1%), black men (-7.1%), and black women (-4.5%). While the reasons for this difference in weight loss response are unclear, social and environmental factors may play a significant role. Some key factors that influence obesity and related health behaviors, and that could be better addressed in lifestyle interventions, are social and cultural influences affecting body image, dietary patterns (e.g., diets high in energy-dense foods), and engagement in physical activity and sedentary behavior [4,5]

Social network theory and analysis is an innovative and valuable framework for understanding the complex characteristics of a person’s social context, the effect this has on health behaviors and outcomes, and to inform the design of “network interventions” [6,7]. The characteristics of a person’s social network--meaning the people (e.g., family, friends, acquaintances) they are connected to and their web of interpersonal relationships--can influence health by shaping access to information, opportunities, social norms, and social influences, thereby enabling or constraining behavior [8]. Social networks have been documented to influence individuals’ obesity risk, diet, and physical activity, as well as individuals’ capacity to change these behaviors [9,10]

A gap in this literature is understanding how relationships between social networks and obesity may differ based on racial and ethnic differences in social norms and weight related behaviors. These insights are needed to inform tailored network intervention strategies for populations with the greatest need for effective obesity treatments. There is evidence of race/ethnic differences in the structure of people’s social networks. On average, non-Hispanic black adults have smaller personal social networks than their non-Hispanic white counterparts [11] but larger than adults of Hispanic origin. Similarly, Hispanics and non-Hispanic black individuals have networks comprised of more family than friend members and more contact with those family members [12]. Overall, personal social networks that are smaller in size often have a greater proportion of members who know each other (referred to as high ‘network density’), enabling a more rapid spread of information and behaviors amongst members and creating stronger social norms. The downside to small, dense networks for individuals with obesity is that the network members are often *homogenous* (i.e., similar to each other and the focal individual). Thus, there tends to be increased exposure to network members with obesity, which may normalize obesogenic behaviors and constrain exposure to alternate practices [13]. Lifestyle interventions that incorporate network intervention strategies could consider and address these social phenomena.

Social support and social undermining are two key network processes that are likely important to address in obesity treatments. Social undermining, distinct from the absence of social support, is defined as negative social interactions that attempt to hinder goal attainment, whether intentional or not [14–16]. To date, few studies have quantified the sources (family, friends, and coworkers), forms (undermining eating vs. physical activity behaviors), and impact of undermining on weight over time. In one study evaluating an employer-sponsored weight loss program Wang et al. measured program participants’ perceived exposure to both social support and social undermining from family, friends, and co-workers, and assessed their influence on study participants’ weight [17]. They found that friend and co-worker support for healthy eating, alongside family support for physical activity, were all significantly associated with reduced weight at two years. Additionally, while family support for healthy eating did not influence participants’ weight, family undermining of healthy eating was associated with actual weight gain. Similarly, in a 12-month behavioral lifestyle intervention trial, Winston et al. demonstrated that non-Hispanic Black and Hispanic adult participants who retrospectively recalled supportive social network members had more significant weight loss at the end of the study than participants who solely experienced social undermining of their behavioral goals [18]. Participants who only experienced social undermining throughout the trial gained rather than lost weight. Weight gain was also associated with having social network members with obesity living in the home, larger network size (i.e., number of individuals), and having ongoing episodes of interpersonal conflict within one’s social network. Overall, the evidence shows that social networks, including their structure, composition, and functions, are significant predictors of obesity and weight loss. Network intervention strategies should be incorporated in weight loss interventions, which leverage or address, and ultimately intervene on, these social phenomena. Still, research is needed to tailor these strategies for Black and Hispanic individuals who may have different social network characteristics, social norms, and face disproportionate burdens of obesity risk.

The overall aim of the Reducing Obesity Using Social Ties (ROBUST) study is to evaluate the feasibility and efficacy of addressing social network processes that may impact optimal weight loss among individuals enrolled in an evidence-based 24-week lifestyle intervention modeled after the Diabetes Prevention Program. This includes addressing social undermining and activating processes associated with communal coping [19] (i.e., communication, social support). More specifically, our goal is to:

1) Culturally adapt the intervention to the needs of the target population while also accounting for gender-based differences regarding engagement in lifestyle interventions and social network characteristics.
2) Examine the feasibility of engaging up to two personal network members of the primary program participants in skill-building sessions to reduce social undermining and increase communal coping, to facilitate the primary participant’s behavioral lifestyle goals.
3) Test whether the social network-enhanced intervention leads to more significant weight loss among participants randomized to the intervention arm, compared to those in the control arm (with no social network component).
4) Evaluate if there are spillover effects of the social network-enhanced behavioral lifestyle intervention on the health behaviors and weight of the participating social network members.
5) Explore if study participants’ social network phenotypes (i.e., social networks with particular structures, compositions, or functional characteristics) predict differences in weight loss over time regardless of intervention arm.

## Materials and methods

### Trial design

The study is a small-scale randomized controlled trial of a social network-enhanced lifestyle intervention compared to an individual-level lifestyle intervention (control group) in 132 adults who self-identify as Black or Hispanic and have obesity (BMI ≥ 30 kg/m^2^). We hypothesize that the social network-enhanced lifestyle intervention will be feasible and acceptable to participants, and will reduce interpersonal barriers to weight loss, such as social undermining while enhancing social support and healthy social norms within the network. The study is comprised of two phases:

**Phase 1: A cultural adaptation of the intervention phase** using semi-structured interviews and a user-centered design iterative process among a subgroup of the target population.
**Phase 2: An implementation and evaluation phase** where the feasibility, acceptability, and early efficacy of the social network-enhanced lifestyle intervention on weight loss will be measured.

**Fig 1:**
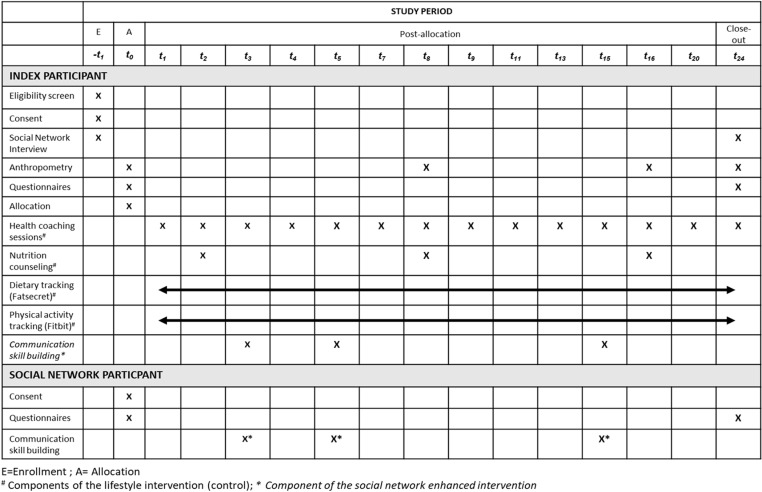
Schedule of enrollment, intervention, and assessments for study participants.

This study protocol adheres to the guidelines of the Standard Protocol Items: Recommendations for Intervention Trials (SPIRIT) (Fig 1, S1 SPIRIT checklist) [20]. Eligible participants who receive their general primary and specialty care at four clinical practice sites within the same healthcare system will be screened via the electronic health record (EHR). After verifying eligibility, participants will be contacted on at least three separate occasions via EHR patient portal messages, email, or telephone and introduced to the study. Interested participants will be electronically consented. More information about the informed consent process can be found in the study protocol **(S2).** The study employs a 1:1 block randomization scheme with varying block sizes and no stratifications. Allocations are performed through REDCap by one research staff member who has access to the randomization module. While the study investigators, statistician, and data analyst will be blinded to the study assignment, the same will not be possible for the health coaches, nutritionists or the study participants.

Consented participants will receive an electronic secure link to complete the self-administered baseline questionnaire and a virtual appointment with a research staff member to conduct the social network interview. Upon completing the social network interview, participants randomized to the control arm will be scheduled for an in-person enrollment visit (t_0_). In contrast, participants randomized to the intervention arm will be given a maximum of two weeks to recruit two social network members into the study. Participants unable to engage a social network member will remain in the intervention arm. Index participant will be scheduled for the in-person enrollment visit within 2 weeks of consenting. The index participants’ social network members who enroll in the study will also complete an online self-administered baseline questionnaire.

Both intervention and control index participants will receive study materials, be oriented to the use of the physical activity and dietary tracking smartphone applications, and complete anthropometric measurements at the in-person baseline enrollment visit (t_0_). Both groups receive the same schedule of health coaching and dietary counseling sessions, with four sessions (t_0_, t_8_, t_16_, and t_24_) conducted in person and the remaining eleven sessions conducted virtually. The content of the coaching and dietary sessions for both arms are identical except for t_3_, t_5_, and t_15_. Participants randomized to the intervention will have their selected and consented social network members join them for the interpersonal skill-building sessions (t_3_, t_5_, and t_15_). After all sessions, all index participants (intervention and control) and their consented social network members (intervention only) will complete an online close-out questionnaire (t_24_). All index participants will also complete a social network interview and anthropometrics measurements at the last in-person coaching session (t_24_).

Written ethical approval was received from the BRANY Institutional Review Board (# 23-08-702-380) on December 18^th^, 2023. Any changes to the existing study, including changes to eligibility criteria, outcomes, or analyses, that may warrant the submission of a new protocol or an amendment will be submitted to BRANY IRB. The ethics committee has requested at least an annual review of the protocol and data, which will include the study enrollment census and any reports of adverse effects. The ethics committee will be notified of any severe adverse effects within 24 hours. Specific procedures for reporting protocol amendments and modifications to all relevant parties can be found in S2.

### Social network enhanced lifestyle intervention

**Fig 2:**
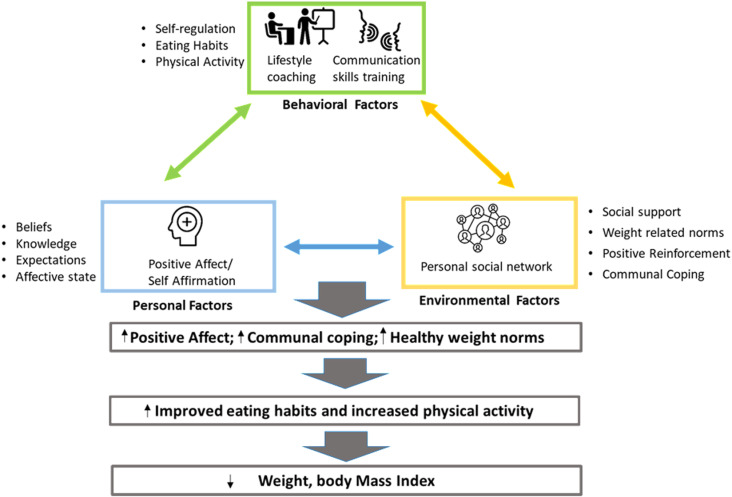
The ROBUST intervention’s conceptual framework is grounded in the Social Cognitive and Social Network Theory.

Our study is informed by the social cognitive theory (SCT), which posits that individual behaviors are shaped by a dynamic interaction between personal factors (e.g., beliefs, skills, resources), behavior, and environments. This interaction is demonstrated by the construct called Reciprocal Determinism. As shown in (Fig 2), personal factors, environmental factors, and behavior continuously interact through influencing and being influenced by each other. An intervention must target all three elements to invoke lasting change in behaviors. The ROBUST intervention builds on the foundational principles of the DPP programming by specifically targeting environmental factors: specifically, social elements of the environment that increase social support, reduce social undermining, and increase collaborative problem-solving (communal coping) within intervention participants’ naturally occurring personal social network [21]. Personal (beliefs, knowledge, expectations, and affective state) and behavioral factors (dietary and physical activity regulation habits) will be addressed through intervention components that are delivered to all participants (intervention and control). By intervening on and measuring key variables at the individual and interpersonal level, we have married the SCT to Social Network Theory (SNT). SNT elucidates the role of social relationships and social structure in transmitting information, channeling interpersonal influence, and enabling attitudinal and behavioral change.

Cultural adaptation of an intervention involves careful consideration of the needs of the individuals for whom the intervention is being developed and a meaningful collaboration between researchers and the target population throughout the process. This process was structured using Barrera’s framework for the cultural adaptation of interventions, which categorizes the process into the following five steps: 1) information gathering, 2) preliminary adaptation design, 3) preliminary testing, 4) refinement, and 5) final design. During the information gathering and preliminary adaptation stage, we purposedly sampled adults 18 years of age or older with a body mass index of 30 or more who self-identified as non-Hispanic Black or Hispanic and who had previously participated in a clinical behavioral weight loss program at one of the study sites for the trial. An interview guide was used to facilitate the individual interviews performed by the same trained research coordinator. Interview guide areas included: 1) the positive and negative influences that social networks have on a person’s behavioral attempts to lose weight; 2) gender, racial, and ethnic differences related to the size and influence of their social network; 3) gender, racial and ethnic differences related to weight loss attempts, methods, and success; and 4) practical approaches to informing and engaging social network members into a research study where they are not the primary participant but instead a support partner. After information gathering, the tailoring of intervention materials and the approach were presented in individual semi-structured interviews with five participants who previously participated in the information-gathering phase. Participants were invited to read the components of the intervention manual, review recruitment materials, and further elaborate on the approach to recruiting social network members. An iterative refinement process was used to finalize the three interpersonal skill-building sessions.

The ROBUST intervention also includes the addition of interpersonal skill-building sessions designed to elicit a communal coping process [21] among the index participant and their network members. In the week three session (t_3_), the health coach will review the evidence that overall health and the risk of obesity are very interconnected with those around us, and thus is a challenge that can be more effectively addressed collectively. Specifically, family and friends can influence health behavior, weight, and beliefs about what a ‘normal’ body size entails by providing support and cues. During the session, the team will watch a video that portrays a day in the life of an adult trying to lose weight but is exposed to three scenarios of social undermining by family and friends. As most of the undermining is unintentional, the video focuses on recognizing social undermining, communicating the impact to friends and family members, and how to counter it as a mutual goal. At the end of the session, participants and their social network members will be allowed to select from one of five “team” building activities to complete before the next skill-building session. Subsequent skill-building sessions will improve commitment, communication, and encouragement of health behaviors. Fundamental techniques include role modeling, behavioral rehearsal, positive reinforcement, and assignments. Participants will be taught effective ways to express positive feelings (e.g., recognizing success, expressing gratitude), making requests (e.g., going to the gym together, helping with transportation), and expressing negative feelings (e.g., feeling disappointed) related to health behavior changes. Effective communication and support skills training will be integrated throughout the intervention by having participants and social network members practice when and where these skills could be most helpful in their daily environments. The components of the interpersonal skill-building sessions are listed in Table 1.

**Table 1:** Content summary of ROBUST intervention sessions at weeks 3, 5 and 15.

Session	Content	Post Session Activity
Week 3	Session objectives include learning about: 1) How the community and the people around an individual effect their body weight; 2) the health effects of having excess weight; 3) How to spot when your health goals are being undermined by others; and 4) the 3 steps to approaching weight loss as a team, by putting the “We” in Weight loss	Choose one of five activities to complete by week 5: -Team cooking - Fitness challenge - Shared gratitude journal - Family health portrait
	Watch ROBUST social undermining video. The video walks participants through three scenarios of unintentional undermining caused by the family and friends of a fictional character who is trying to lose weight.	
	Introduction of the three elements of communal coping: orientation, communication regarding the stressor (i.e., excess weight), and cooperative action to address the stressor	
Week 5	Session objectives include learning about;1) the lifestyle recommendations for a person living with obesity; 2) How to use team coping skills to improve team communication; and 3) How to solve problems as a team.	Set a Team SMART goal and create an action plan inclusive of how to address challenges
Week 15	Session objectives include learning about: 1) How to identify both negative and positive social cues. 2) How to replace negative social cues with positive ones and 3) How to approach social cues as a team	Social Cue Awareness activity select 3 social cues that have impacted personal weight loss, create an action plan that describes how to face each social cue as a team.

### Lifestyle intervention (control)

**Fig 3:**
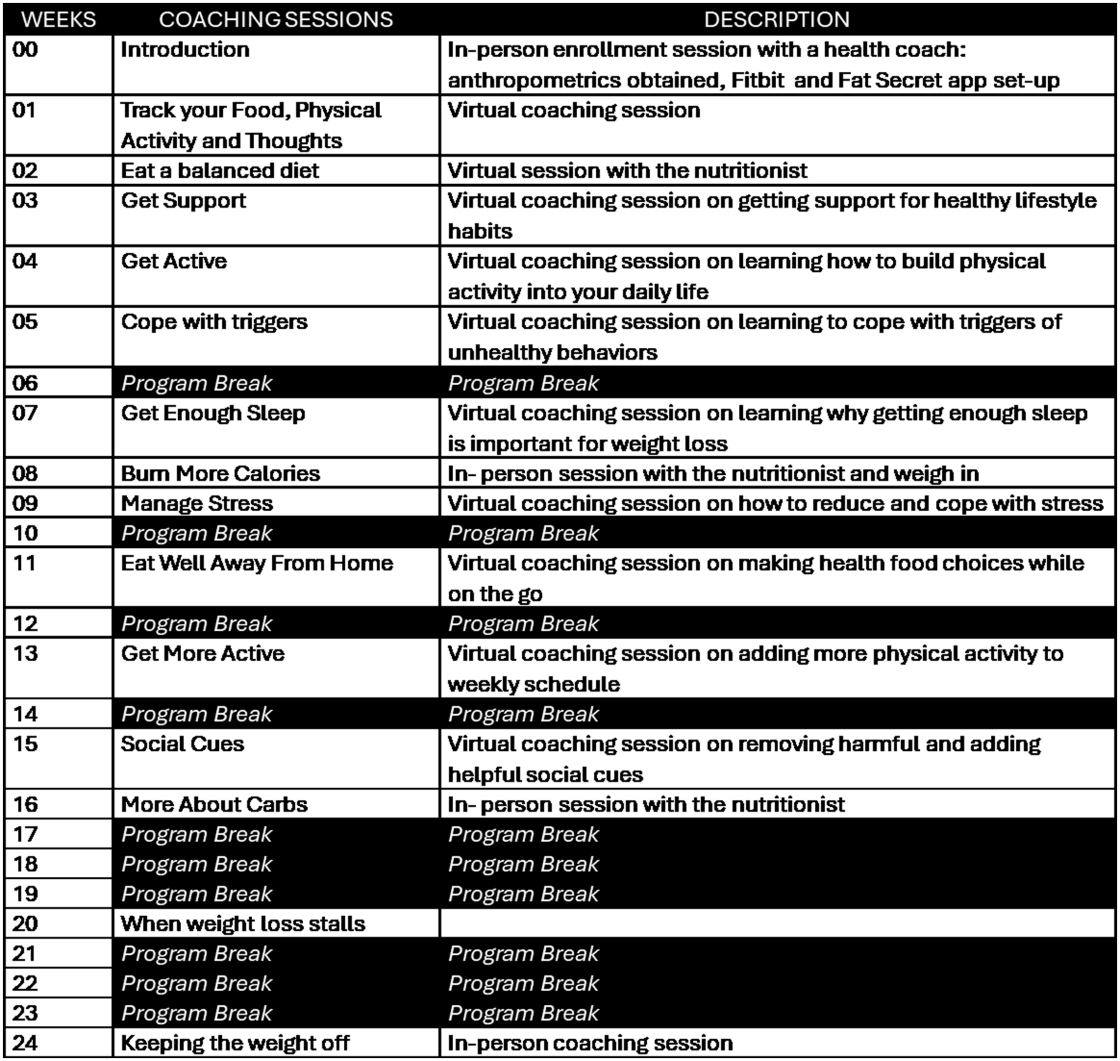
Schedule of coaching sessions conducted over 24 weeks.

The core components of the lifestyle intervention are grounded in standard behavioral change methods, including goal setting, problem-solving, relapse prevention, and motivational interviewing, based on the evidence-based Diabetes Prevention Program (DPP) [22,23]. Fig 3 depicts the DPP lifestyle sessions delivered as one-on-one virtual coaching sessions. All participants, regardless of study arm, receive the same eleven core sessions.

At the t_0_ and t_1_ study visits, participants will be taught the self-regulation skills of tracking their weight, physical activity, and dietary intake. The latter will be monitored using the FatSecret smartphone application. FatSecret works well across electronic platforms, it has been used successfully in previous research, and participant data is readily available to research teams by setting up a professional account to which study participants connect their data [24,25]. There is no cost to use the basic features; for purposes of the study, a 6-month premium plan will be purchased for each participant, providing access to the app’s calorie-based balanced meal plans, shopping list, and recipes.

Participants will be instructed to complete a 3-day food record before each dietary counseling session (t_2,_ t_8, and_ t_16_). In preparation for the first dietary counseling session, the food records and baseline questionnaire data will be reviewed to understand the participant’s dietary preferences and potential barriers to behavior change (e.g., food accessibility). At visit 2, participants will be provided a calorie level individualized to their age, sex, height, weight, and activity factor with a 500 kcal/day deficit for weight loss using the Mifflin-St Jeor formula. A 7-day meal plan will be selected based on the daily caloric goal and scheduled in the app’s diary. Participants will be encouraged to use the app’s shopping list for each meal plan created. If the meal plan does not align with the participant’s dietary preferences, participants will be trained to utilize app functions to ensure they meet their daily caloric goal when deviating from the meal plan foods. The meal plan will be scheduled for six weeks and be readjusted at the week eight dietary counseling session.

Prior to week 8 and week 16, the food records and data collected from previous sessions (e.g., session notes and self-reported weight) will be reviewed to inform the in-person dietary counseling session. Barriers to following the meal plan and utilizing FatSecret will be addressed at the visit. At week 8, participants will engage in a food portion activity using food models to assess participants’ accuracy when estimating amounts consumed. During the week 16 visit, participants will engage in an activity focused on differentiating low versus high fiber per serving foods and increasing individuals’ dietary fiber intake at each meal. A new individualized meal plan will be prescribed based on the participants’ weight obtained at both assessment visits.

Similarly, individualized physical activity plans will be made based on the current activity level, and weekly incremental increases in moderate physical activity until at least 150 minutes per week is achieved. Participants will be given the FitBit Inspire fitness tracker and instructed to wear it at least nine hours a day during regular activity days and to enter purposeful physical activity that is not automatically detected. Lastly, participants will be counseled to weigh themselves at least once a week using the scale given to them by the study. Study personnel will provide standardized written instructions on conducting home weights and demonstrate proper scale usage at the baseline study visit.

### Study setting and participants

Participants in Phase 2—the implementation and evaluation phase—will have to meet the same age, self-identified race, ethnicity, and BMI criteria as Phase 1 participants--the cultural adaptation phase. In addition, Phase 2 participants will have to have access to a smartphone and be willing to identify up to two social network members to engage in the study if randomized to the social network enhanced lifestyle intervention arm. Index participants will be excluded if they are actively enrolled in another weight-loss program, use weight-loss medications, have a history of bariatric surgery, or are planning to have surgery in the coming year. Additional exclusion criteria include advanced medical illness, dementia, recent hospitalization, injury or pregnancy that inhibits regular physical activity, contraindications to exercise based on lack of clearance from a healthcare provider, unresolved food insecurity, or inability to speak English or Spanish.

Social network members invited to participate by the index participant must be 18 years or older and have access to the internet or a smartphone. Social network members who need help to speak English or Spanish well will be excluded.

### Recruitment

Recruitment will occur at four ambulatory practice sites affiliated with an urban academic medical center. Potential participants will be identified via a data search of the electronic health record (EHR) system using the eligibility criteria of race, ethnicity, body mass index of 30 or higher, and having at least one visit to the participating practice in the last 12 months. Study staff will contact the primary medical provider to verify that the participant can participate and provide the names of patients from their list who should not be contacted for study participation. Participants will be contacted via phone call, email, or EHR message based on their indicated preferences for communication in the electronic health record. Recruitment strategies will include posting participants’ flyers within the clinical sites, distributing them at community health fairs, and sending direct emails to providers promoting the study. Recruitment of social network members will be done solely by the index participant, and they will be provided with a script with suggestive language developed by the study team. Study recruitment commenced in April 2024. It is expected to be completed by September of 2025 with the last participant follow-up conducted in March of 2026.

### Training and supervision

The study staff will undergo extensive protocol training, which includes weekly team training sessions for two weeks to ensure competency of the study protocol and proper use of study materials. Training includes role-play and personal tracking of dietary and physical activity habits to troubleshoot technology-based challenges. The health coaches and nutritionists all primarily have undergraduate or graduate degrees in nutrition, behavioral psychology, exercise physiology or public health. Coaches are trained in the core competencies of motivational interviewing and behavior change theory. Supervising the social network interviews, coaching, and dietary counseling sessions occurs through live observations or review of audio recordings by the senior investigators (EP, KDLH). During routine weekly and monthly team meetings, staff receive feedback regarding implementation fidelity.

Study staff are also trained in using physical activity and dietary tracking applications. Training videos on how to use the applications created for the study participants are available on the study website platform and will serve as training videos for the staff.

### Social network interview

Before the first in-person baseline session, participants will complete a personal social network interview with a trained study staff member. The interview questions are programmed in Network Canvas, a free personal social network interview and data-management software [26]. The interview includes four phases of questions to elucidate information about participants’ social networks that are commonly used in personal network measures [27]. The interview begins with a personal network name generator, where participants are asked to list the names (first name and last initial, or nicknames) of 15 adults who are important to them and with whom they had contact in the past six months. First, participants report on the characteristics of each nominated network member, including their social role (family, friend, coworker, etc.), socio-demographics (gender, age, race/ethnicity, marital status), and perceived weight status (using Stunkard’s body shape silhouettes) [28]. Second, participants report on their relationships with the named social network members, including frequency of contact, closeness, and living proximity. Third, they are asked questions about the relationship between each unique pair of network members to assess their personal network structure. Finally, their personal social network—comprised of the network members they nominated and the social ties among them—is visualized in the Network Canvas software and used to prompt participants for qualitative feedback on three key features of their network: (i) people who provide social support, (ii) people who are involved in conflict and undermining, and (iii) people who are currently trying to lose weight. The latter prompts are open-ended, and participants’ responses are audio-recorded as qualitative data.

### Data collection

The data collection plan for this study will utilize REDCap to capture all treatment, efficacy, and adverse event data on all enrolled participants. Data on index participants’ social networks will be collected using Network Canvas software (25). Data will be collected, managed, and analyzed throughout the project according to our data safety and monitoring plan and reviewed bi-annually by the appointed data safety monitoring officer (MS). All participant data will be managed and stored on secure servers at Weill Cornell Medicine. All identifiable information in the participant interviews (e.g., names and locations) will be removed before analysis. As in any study, missing values may occur in outcomes due to study drop-out, withdrawal or lost to follow-up. Participants are free to withdraw from participation in the study at any time upon request. An investigator may discontinue or withdraw a participant from the study for the following reasons: a) Pregnancy; b) Significant study intervention non-compliance; c) if any clinical adverse event (AE) or other medical condition or situation occurs such that continued participation in the study would not be in the best interest of the participant; d) if the participant meets an exclusion criterion (either newly developed or not previously recognized) that precludes further study participation; e) participant lost to follow-up after several attempts to contact subject to schedule study visit. The reason for participant discontinuation or withdrawal from the study will be recorded. Subjects who sign the informed consent form and are randomized but do not complete any aspect of the t_0_ assessment will be replaced.

A thorough investigation of mechanisms for missing data will be carried out. We will also aim to prevent missing data by training our research staff to collect high-quality data with standardized protocols and ongoing quality controls. The primary outcome of feasibility and acceptability will be evaluated for developing a more extensive trial using the a priori criteria in Table 2**. ‘**Recruitment’ will track the number of participants approached, screened, found eligible/ineligible, declined participation, and their reason for declining. ‘Data collection’ will be followed based on the quantity of missing data and the number of participants lost to follow-up. ‘Integrity of study protocol’ will be used to identify the feasible and acceptable dose of the intervention, as well as treatment fidelity. Session attendance will be measured as a marker of adherence, with a benchmark of attending at least 75% of the coaching/assessment sessions for both index participants (11 out of 14) and social network members (2 out of 3). Treatment (intervention) fidelity will be assessed in three ways. The first is a checklist the coach will complete at the end of each session. This checklist will also remind the coach about the active ingredients to be delivered during the session. The second method is a monthly review of a random sampling of 10% of the coaching sessions. Points will be given for each required treatment element delivered correctly and subtracted for any contamination elements. The coach will be retrained if the average fidelity falls below 90%. The third is by tracking the session duration in minutes.

**Table 2:** A priori Feasibility Criteria.

Recruitment/ Retention	Number of index participants and social network members assessed for eligibility, excluded, consented, lost to follow up, and withdrawals.
**Data Collection**	Collect data on 100% participants (ego and social network members) at T0 (baseline) and 85% at T24 weeks
**Treatment Fidelity**	Index participants and social network members will attend 75% of their coaching sessions. At least 9 out of 10 (90%) items from the intervention checklist will be implemented as written. Session duration will not exceed the allotted time by more than 10 minutes.
**Technical Issues**	<10% of technical issues with the utilization of the FatSecret app.
**Satisfaction**	>=90% report excellent or very good satisfaction with the intervention

The coach will assess enactment fidelity at the beginning of each coaching session using a simple yes/no checklist. ‘Technical issues’ will determine the feasibility of using the FatSecret web-based application. We will ask open- and closed-ended questions of index participants and the consented social network members regarding satisfaction with various aspects of the program (e.g., telephone/ virtual delivery of the intervention, skills gained). Each item will be rated on a Likert scale, with higher scores indicating greater program favorability. Open-ended questions will be used to evaluate the intervention’s acceptability and which intervention components could be improved and which are not acceptable.

The study will also assess the impact of the social network-enhanced intervention on key social network processes (secondary outcomes), and their role in reducing dietary caloric intake and increasing physical activity, resulting in goal weight loss. Variables of interest and the stated outcomes will be measured as follows:

#### Communal Coping.

will be measured using McMaster Family Assessment Device (FAD) subscales for communication (6 questions) and collaborative problem-solving (5 questions) in the index participant [29]. FAD scores are an individual’s perception of their family’s functioning. For this study, participants will be instructed to think about how well the statements (e.g., “*We try to think of different ways to solve problems”*) describe the relationships of their closest family and friends. Scores for individual items and dimensions range from 1 to 4, with a higher score indicating poorer functioning. Adequate test-retest reliability (.66-.76) and concurrent validity have been reported.

Communal coping will also be assessed at the personal network level, by measuring which nominated personal network members participants “talk to about health problems”, and then computing the proportion of their network members engaged in health communication.

#### Social support.

will be measured with a modified version of the Social Support for Diet and Exercise Behavior Scales [30]. The scales consist of two surveys that assess respondents’ perceptions of positive and negative social support (e.g., undermining) for eating behaviors (10 items) and exercise habits (13 items) from family members and friends, respectively. Respondents rate the frequency with which family members and friends had done or said what was described in the item during the previous three months on a 5-point scale, ranging from 1 (*none*) to 5 (*very often*). The eating behaviors survey produces two subscales (Encouragement and Discouragement), and two subscales are calculated from the exercise habits survey (Participation and Rewards and Punishments).

For this study, we made two minor modifications to the survey subscale items. First, we did not separate subscales for family members and friends. Second because the study’s primary objective is to examine participant characteristics associated with their perceptions of positive and negative social support for eating and exercise, we wanted one of the two exercise habits subscales to capture negative support while the other represents positive support solely. Third, we modified the 5-item Discouragement for Healthy Eating subscale by removing one item (“*got angry when I encouraged them to eat low salt, low fat foods”*) because it was not conceptually linked with the remaining four items, which represented unhealthy actions and behaviors among friends and family, such as buying or eating unhealthy foods in front of the respondent, specifically: *“ate high fat or high salt foods in front of me”; “refused to eat the same foods I eat”; “brought home foods I’m trying not to eat”; and “offered me foods I’m trying not to eat.”* These modified versions have maintained acceptable reliability in other similar studies. (Cronbach’s alpha =.73 and .80 for the modified subscales relating to friends and family members, respectively) [31].

Social support and undermining will also be assessed at the personal network level. Social support is measured by having participants report whom among their nominated personal network members *“helped or encouraged them to have a healthy lifestyle: for example, to eat healthy foods or be active*”, which is used to compute the proportion of participant’s network members that provide health support. Participants also report on whom among their nominated social network members “*made it difficult for them to have a healthy lifestyle: for example, to eat healthy foods or be active”,* which is used to compute the proportion of their network members who are health barriers.

#### Weight-related norms.

in the personal network will be measured from the perspective of the index participant using the Stunkard figure rating scale. The commonly used Stunkard’s figure rating scale comprises a series of nine male or female figure drawings of increasing body size. Figural stimuli have been used in psychological research to assess current body size in adults [32,28]. Index participant’s perceived weight status of each social network member will be used to compute the average weight of their network members, and the proportion of their network perceived to have obesity.

#### Positive affect.

will be measured in index participants using the 10-item positive affect subscale of PANAS [33]. Composite scores range from 10 to 50, with higher scores representing higher positive affect levels.

#### Dietary intake and quality.

will be measured in two ways. First, the Block Fat, Fruit-Vegetable-Fiber Screeners will be used to assess frequency [34]. The Fruit–Vegetable–Fiber Screener is a 10-item instrument that includes the main sources of fruits, vegetables and fiber in the US diet. It provides a rapid assessment of fruit/vegetable servings, dietary fiber, vitamin C, magnesium and potassium intake. This Screener correlates well with fruit and vegetable servings (0.71); validation studies demonstrated that this Screener could identify adults with low intakes of vitamin C, fiber and potassium. The 17-item Block Dietary Fat Screener includes the major sources of fat in the US diet and efficiently estimates total fat, saturated fat and cholesterol intake. Results from a nationally representative survey of middle-aged and older adults indicated that fat intake estimates using the Block Dietary Fat Screener and fat intakes assessed by diet recalls and other food frequency questions had correlations (that is, *r*=0.33–0.60) like other published validation studies [35,36]. In addition, among middle-aged women, the Fat Screener correlated well with the total grams of fat calculated from 4-day diet records (*r*=0.58).

Three-day food records (FRs) captured via the FatSecret App will estimate mean intakes of energy (kcal), carbohydrates, protein, fat, and fiber per day. To capture day-to-day variations, participants will be asked to keep non-consecutive 3-day FRs, including two weekdays and at least one weekend day, at least three times throughout the study. Subjects will be trained in portion size estimation and encouraged to record all intake as much detail as possible.

#### Physical activity.

will be subjectively assessed using the short version (9 items) of the International Physical Activity Questionnaire (IPAQ) [37]. The instrument has reasonable measurement properties for monitoring physical activity among 18- to 65-year-old adults in diverse settings. Objective measures of physical activity will be obtained using a Fitbit Inspire 3 (Fitbit Inc., San Francisco, CA). Fitbit is a wrist-worn, tri-axial accelerometer with continuous optical heart rate monitoring. It syncs with the Fitbit smart phone app. It yields physical activity metrics including steps, calories (converted to intensity in metabolic equivalent minutes (METs) by multiplying Fitbit calories by body weight and dividing by time), active minutes (in bouts of ≥10 min), sleep, and hourly activity (frequency measured by the number of hours between 09:00 and 17:00 daily when ≥250 steps or significant arm movements were registered). Fitabase (an independent research data platform (Small Steps Labs, LLC, San Diego, CA) will be used to access minute-by-minute Fitbit data.

#### Weight.

change will be measured as a mean percentage reduction in baseline body weight and body fat at week 24. Training staff will measure weight and percent body fat at four time points (weeks 0, 8, 16 and 24) using a bioelectrical impedance analysis scale (Tanita DC-430U).

### Data and safety monitoring

Overall, the intervention and measurement protocols pose minimal risk to the index participants and their participating social network members. Because of this low-risk status, the data safety monitoring plan (DSMP) for this trial focuses on close monitoring by the principal investigator in conjunction with a safety officer and prompt reporting of excessive and severe adverse events to the NIH and the respective ethics committee. Safety reports will be sent to the study statistician and the safety officer at a pre-determined frequency based on the data type. Details of the corresponding DSMP and the data dissemination plan can be found in S3. A description of plausible expected adverse events and stopping rules are described in the protocol **(S2).**

## Statistical considerations

### Sample size

Phase 1 will include a convenient sample of participants who meet the eligibility criteria. As standard in qualitative methodology, the exact number of subjects needed will depend on when saturation of ideas has emerged from reviewing interview transcripts. Based on our previous experience with similar iterative design sessions, we anticipate that saturation will be achieved with around 20 adults, to recruit racial, ethnic, and gender-diverse participants equivalent to the planned distribution for phase 2 (60% women and 50% Hispanic).

The sample size for Phase 2 has been guided by our preliminary data which demonstrate that the network-level barrier of social undermining, an understudied social construct, significantly influences weight outcomes. There was an 8-pound difference (3.7%) between participants who experienced social undermining versus those who did not in the SCALE trial [38]. To our knowledge, no studies have targeted this construct using communal coping [21]. In comparing baseline mean scores across published studies, we have noted a 3-point difference (3.2± 4.9) in the mean score for undermining healthy eating habits between non-Hispanic Whites (9.6 ±3.9) vs. non-Hispanic Blacks (12.8±4.9). Thus, setting this as a meaningful difference in social undermining scores between the ROBUST and control group, with a two-sided significance test where α is 0.05 and power of 90%, a sample size of 132 participants would be needed, accounting for 15% attrition. The attrition rate is based on a recent lifestyle intervention study completed by the study lead investigator, where most health coaching was delivered virtually or by phone. Compared to remote or group-based sessions, individualized coaching sessions afford participants greater flexibility and are thus likely the reason for a lower-than-expected loss to follow-up rate of 13%. We estimate that 800 individuals must be screened to enroll 132 index participants.

Additional sample size and power estimate calculations were conducted to account for the secondary weight loss outcome. Numerous studies have translated the Diabetes Prevention Program (DPP) to “real world” settings to reach participants with low socioeconomic status and diverse races and ethnicities. In one review of 16 studies that translated the DPP protocol in four distinct environments--hospital outpatient, primary care, community, and work/church-- the proportion of participants who met the 5% weight loss goal in primary care and work/church settings ranged from 25–48% [39]. On average, weight loss in real-world DPP translation studies is 4%. With a sample size of 132 participants, we will have more than sufficient power (90%) to detect an 8-pound difference between the intervention and control group. Additionally, we can detect a minimum weight loss difference between the two groups of 6 pounds with 82% power.

To explore personal social network homophily (i.e., similarity between the index participant and their social network members), we will evaluate similarities in index participant self-reported weight, healthy eating, and engagement in physical activity, and their perceptions of the weight/behaviors of their social network members. This will be assessed at baseline and close-out and will compute if the extent of this similarity changes over time. Similarities will be measured using social network indices for homophily, and repeated measure correlation [40].

Feasibility will be assessed based on rates of recruitment, study conduct (intervention and enactment fidelity), retention and acceptability of the study. Descriptive statistics will be used to summarize recruitment rates. The screening-to-enrollment (STE) ratio will be defined and calculated as the proportion of people who consented to those for whom screening was attempted. An STE ratio will be calculated for index participants separately from their network member. The reasons for declining participation will be grouped into categories and the proportion described.

Adherence to several aspects (session attendance, dietary, physical activity and self-weights) of the intervention will be tracked. The proportion of coaching sessions attended by the index participants and social network members across the 24 weeks will be calculated separately. Additionally, a tally of each self-regulation behavior (total # of days weighing, total # of days self-monitoring dietary intake, total # of days wearing fitness tracker). Treatment fidelity will be summarized as an average of the proportion of treatment elements delivered correctly. A correlation (Pearson) analysis between the self-reported checklist and objectively measured checklist will be conducted. The retention rate will be defined as the proportion of participants who complete the 24-week assessment among those who completed the baseline assessment. Sample sizes and the proportion of missing data will be calculated for each measure collected as baseline, follow-up visits and study completion. Chi-Squared tests (or Fisher’s exact tests) and two-independent-sample *t*-tests (or Wilcoxon rank-sum tests) will be used, as appropriate, to examine whether baseline characteristics predict retention and to identify differences in dropout rates between the intervention and control group. The qualitative satisfaction data will be content analyzed. Two study personnel will generate a priori codes corresponding to the interview guide (e.g., reactions to enrolling network members). One study personnel will apply the coding scheme to each interview summary, and then the two will formalize agreement or disagreement regarding the derived codes.

Summary statistics, histograms, and scatter plots of the measured covariates and outcomes for outliers and data trends will be examined. Analyses of differences between those completing and not completing the study and the within-group changes during the intervention will be performed according to modified intention-to-treat principles. Multivariable linear regression (MLR) models will be used to examine the relationship between changes in the social network processes and exposure (the social network lifestyle intervention vs. the lifestyle intervention), adjusted for covariates selected from univariate analyses; potential covariates for MLR are categorized by theoretical construct, as shown in the table below. We will also explore the effect of gender by including a three-way interaction (gender*social support*weight loss), although we recognize that we might not have enough power to detect such interactions.

To further explore the effect of the intervention, we will do sensitivity analysis: (1) Multivariable linear regression using weight loss as continuous outcome variables (a change in weight and percent weight change), adjusting for baseline covariates distributed differently between intervention groups; (3) test interaction terms between treatment group and other covariates; and (4) examine completers only.

We will evaluate similarities in weight and related behaviors between the index participant and their respective participating social network members at baseline and close-out and test if similarities increase over time. Similarities will be measured using a repeated measures correlation to assess variables such as a change in weight among connected members of the social network (i.e., the index participant and the participating member).

Personal network data will be analyzed using social network analysis (SNA) packages in R (e.g., sna, network, igraph) to produce visualizations and statistics that summarize the characteristics of each participant’s network. We will compute the following structural, compositional, and function network statistics for each participant, at both the baseline and final (0, 24 weeks) assessments. 1) *Network structure* will be computed based on *density*, which refers to the number of network ties (who knows who) among their nominated network members, divided by the maximum number of possible network ties, and reflects how densely or sparsely their personal network is connected; and *transitivity*, which refers to the tendency for connections among network members to cluster together (i.e., when member A knows B, and member B knows C, then member A and C also know each other). 2) *Network composition* will be measured by calculating the proportion of network members with specific demographic characteristics (e.g., the % of family, friends, same-age peers, presence of a spouse/partner) and the proportion who are obese/not obese (norms). 3) *Social functions* of the network will be computed based on the proportion of network members that are reported to be close, have frequent contact, and that are health communicators, health supporters, or health barriers. We will conduct exploratory, descriptive analyses to understand the types of network members who provide social connection, support, and barriers. These personal network statistics computed for baseline and 24 weeks as well as change statistics across periods (e.g., change in the % of alters that provide support), will be included as index-participant variables in the multivariate model.

Among all index participants (intervention and control group), we will examine if the network phenotype (defined by latent class analysis) at baseline affects the behavior change process and the outcome of weight loss. We will use LCA to group the study participants by individual variables (age, sex, race/ethnicity, education, marital status, employment, baseline weight and percentage body fat) and personal network variables (living proximity, frequency of contact, closeness, weight norm). As latent class analysis (LCA) can only fit categorical variables, each continuous variable will be divided into tertiles. The advantages of LCA over cluster analysis include the following: latent class analysis is (a) model-based, (b) allows the generation of probabilities for group membership, and (c) tests goodness-of-fit across competing models. Fit indices will be examined to help select the optimal model for the data. Specifically, a better model will be suggested by a lower Akaike information criterion (AIC); a lower Bayesian information criterion (BIC); a significant Lo-Mendell-Rubin likelihood ratio test, which indicates that the more complex model (i.e., model with more patterns) fits the data better than the model with fewer patterns; entropy, the estimate of certainty of classification (ranging from 0 to 1); and meaningfulness or interpretability of the response patterns [41–43]. Linear regression models will be utilized to assess the relationship between latent groups and several outcomes of interest, including changes in physical activity, dietary intake, and weight loss at 24 weeks. All models will be adjusted for sex, age, education, and randomization grouping. All analyses will be performed using SAS 9.4M8 and R 4.4.0.

## Discussion

Much of the prior research on the influence of social networks on obesity has been in cross-sectional or longitudinal studies of non-Hispanic white populations. These approaches may underestimate social networks’ impact on populations with very different network characteristics and dynamics. This study will be one of the few prospective studies to intervene on negative network processes while prospectively characterizing the network and measuring weight and behavior changes in at least two close ties and changes in the network dynamics (the structure, function, and composition). Our approach will provide new knowledge and strengthen previous findings that have been limited in scope.

Although no study is likely to prove causality independently, randomization reduces bias. It also provides a rigorous method to examine the cause-effect relationship between the ROBUST intervention, changes in the desired social network processes, and, ultimately, weight loss. We will apply rigorous measurement techniques using validated instruments and trained study personnel. Our statistical analyses have incorporated a social network analytic approach to understanding the complex selection processes responsible for the social relationships that help shape health behaviors.

Attrition is one of the major causes of treatment failure in obesity. To reduce the burden of group-based in-person or virtual coaching sessions, we have adapted the DPP intervention model to be delivered one-on-one virtually. To ensure that all participants receive the same dose and frequency of the intervention, we will establish a protocol manual, use computer prompts as reminders, and record deviations from the protocol [44]. We will also randomly record individual counseling sessions to gauge adherence to the study protocol.

Engaging personal networks in weight-loss interventions is an area that is under-examined, and we aim to understand the impact of personal network characteristics on participant engagement and dropout. We hypothesize that drop-out among participating network members is unlikely to significantly impact the communal coping process or our ability to capture pertinent data. Our expectation of the ROBUST intervention is that activation of communal coping will have ripple effects throughout the participants’ personal network and reach their social ties beyond just the 1 or 2 network members who enroll in the study. In a formative evaluation study conducted by de la Haye [45] where the communal coping process was similarly triggered by a health risk assessment tool followed by behavioral feedback, 76% of participants who were all low-income non-Hispanic Black adults communicated with others (including family, friends, work colleagues) about their health risk to prompt others to do the same [45]. We will be able to capture these pertinent interactions between the index participant and their network during the personal network assessment conducted at the study closure.

This study proposes the implementation of an original and innovative intervention to address social processes that may hamper optimal weight loss in high-risk groups enrolled in behavioral lifestyle programs. If the results of this study show a significant impact on social support, social undermining, and weight, this would provide new evidence and new avenues to explore deploying/implementing the study on a larger scale and over a longer period.

## Supporting Information

S1 File SPIRIT 2013 checklist: Recommended items to address in a clinical trial protocol and related documents. (DOCX)

S2 File Detailed protocol. (DOCX)

S3 File Data safety monitoring plan. (DOC)
