# Supplementary material for: Reducing Obesity Using Social Ties (ROBUST): Protocol for a randomized control trial of a social network lifestyle intervention
Source: PLoS One. 2025 Apr 16;20(4):e0318990. doi: 10.1371/journal.pone.0318990 (PMC12002803; doi:10.1371/journal.pone.0318990)
Supplement: S2 File — (DOCX) [file pone.0318990.s002.docx]

**TITLE:** **R**educing **OB**esity **U**sing **S**ocial **T**ies (ROBUST): Randomized Controlled Trial

**IRB Protocol #:** 23-02025770

**IND/IDE #:** Not applicable

**Version Date:** 10/6/2023

**Funding Source(s):** National Institute of Diabetes and Digestive and Kidney Diseases

**Participating Clinical sites:**

1. Weill Cornell Internal Medicine Associates

505 East 70^th^ Street Helmsley Tower 4^th^ Floor

New York, NY, 10021

PI: Erica Phillips, MD, MS

1. Weill Cornell Urology

**Upper East Side**
525 East 68th Street
Starr Pavilion, 9th Floor (Starr 900)
New York, NY 10065

PI: Erica Phillips, MD, MS

1. Weill Cornell Urology

**Brooklyn**

**38 6^th^ Avenue**

**Brooklyn, NY 11217**

PI: Erica Phillips, MD, MS

1. Weill Cornell Primary Care at Lower Manhattan

156 William Street, 6^th^ Floor

New York, NY, 10038

PI: Erica Phillips, MD, MS

Table of Contents

[Confidentiality Statement vii](#_Toc145612218)

[List of Abbreviations ix](#_Toc145612219)

[1. Protocol Summary 1](#_Toc145612220)

[1.1 Schema 3](#_Toc145612221)

[1.2 Study Objectives and End Points 6](#_Toc145612222)

[1.2.1 Primary Objectives 6](#_Toc145612223)

[1.2.2 Secondary Objectives 6](#_Toc145612224)

[1.2.3 Exploratory Objectives 6](#_Toc145612225)

[1.2.4 Primary Endpoints 6](#_Toc145612226)

[1.2.5 Secondary Endpoints 6](#_Toc145612227)

[2. Background 6](#_Toc145612228)

[2.1 Disease 6](#_Toc145612229)

[2.2 Investigational Agent/Device, or Surgical Treatment/Method 6](#_Toc145612230)

[2.3 Rationale 6](#_Toc145612231)

[2.4 Risk/Benefit Assessment 7](#_Toc145612232)

[2.4.1 Known Potential Risks 7](#_Toc145612233)

[2.4.2 Known Potential Benefits 7](#_Toc145612234)

[2.4.3 Assessment of Potential Risks and Benefits 7](#_Toc145612235)

[2.5 Correlative Studies Background 7](#_Toc145612236)

[3. Study Design 8](#_Toc145612237)

[3.1 Overall Design 8](#_Toc145612238)

[3.2 Scientific Rationale for Study Design 8](#_Toc145612239)

[3.3 Justification for Dose 8](#_Toc145612240)

[3.4 End of Study Definition 8](#_Toc145612241)

[4. Subject Selection 8](#_Toc145612242)

[4.1 Study Population 9](#_Toc145612243)

[4.2 Inclusion Criteria 9](#_Toc145612244)

[4.3 Exclusion Criteria 9](#_Toc145612245)

[4.4 Lifestyle Considerations 9](#_Toc145612246)

[4.5 Screen Failures 10](#_Toc145612247)

[4.6 Strategies for Recruitment and Retention 10](#_Toc145612248)

[5. Registration Procedures 11](#_Toc145612249)

[5.1 Subject Registration (WCM only) 11](#_Toc145612250)

[5.2 Subject Registration (Sub-sites) 11](#_Toc145612251)

[6. Study Procedures 11](#_Toc145612252)

[6.1 Schedule of Assessments 11](#_Toc145612253)

[6.1.1 Screening Visit 13](#_Toc145612254)

[6.1.2 Treatment Phase 13](#_Toc145612255)

[6.1.2.1 Visit 1 13](#_Toc145612256)

[6.1.2.2 Visit 2 (± X day(s)) 13](#_Toc145612257)

[6.1.3 Follow-up Phase 13](#_Toc145612258)

[7. Study Intervention 13](#_Toc145612259)

[7.1 Study Intervention/Device Description 13](#_Toc145612260)

[7.2 Availability 14](#_Toc145612261)

[7.3 Acquisition and Accountability 14](#_Toc145612262)

[7.4 Formulation, Appearance, Packaging, and Labeling 14](#_Toc145612263)

[7.5 Product Storage and Stability 14](#_Toc145612264)

[7.6 Preparation 14](#_Toc145612265)

[7.7 Dosing and Administration 14](#_Toc145612266)

[7.7.1 Dosing Delays/Dose Modifications 15](#_Toc145612267)

[7.8 General Concomitant Medication and Supportive Care Guidelines 15](#_Toc145612268)

[7.9 Duration of Therapy and Criteria for Removal from Study 16](#_Toc145612269)

[7.10 Duration of Follow Up 16](#_Toc145612270)

[7.11 Measures to Minimize Bias: Randomization and Blinding 16](#_Toc145612271)

[7.12 Study Intervention/Follow-up Compliance 17](#_Toc145612272)

[8. Study Intervention Discontinuation and Participant Discontinuation/Withdrawal 17](#_Toc145612273)

[8.1 Discontinuation of Study Intervention 18](#_Toc145612274)

[8.2 Participant Discontinuation/Withdrawal from the Study 18](#_Toc145612275)

[8.3 Lost to Follow Up 19](#_Toc145612276)

[9. Correlative/Special Studies 19](#_Toc145612277)

[9.1 Laboratory Correlative Studies 20](#_Toc145612278)

[9.1.1 Title – Laboratory Correlative Study #1 20](#_Toc145612279)

[9.1.1.1 Collection of Specimen(s) 20](#_Toc145612280)

[9.1.1.2 Handling of Specimen(s) 20](#_Toc145612281)

[9.1.1.3 Shipping of Specimen(s) (if multicenter) 20](#_Toc145612282)

[9.1.1.4 Site(s) Performing Correlative Study (if multicenter) 20](#_Toc145612283)

[9.2 Special Studies 20](#_Toc145612284)

[9.2.1 Title – Special Correlative Study #1 20](#_Toc145612285)

[9.2.1.1 Assessment 20](#_Toc145612286)

[9.2.1.2 Method of Assessment 20](#_Toc145612287)

[9.2.1.3 Timing of Assessment 20](#_Toc145612288)

[10. Measurement of Effect 20](#_Toc145612289)

[10.1 Response Criteria 20](#_Toc145612290)

[10.2 Duration of Response 20](#_Toc145612291)

[10.3 Progression-Free Survival 21](#_Toc145612292)

[10.4 Other Response Parameters 21](#_Toc145612293)

[11. Data Reporting / Regulatory Considerations 21](#_Toc145612294)

[11.1 Data Collection 21](#_Toc145612295)

[11.1.1 REDCap 21](#_Toc145612296)

[11.2 Regulatory Considerations 21](#_Toc145612297)

[11.2.1 Institutional Review Board/Ethics Committee Approval 21](#_Toc145612298)

[11.2.2 Ethical Conduct of the Study 22](#_Toc145612299)

[11.2.3 Informed Consent 22](#_Toc145612300)

[11.2.4 Compliance with Trial Registration and Results Posting Requirements 23](#_Toc145612301)

[11.2.5 Record Retention 23](#_Toc145612302)

[12. Statistical Considerations 23](#_Toc145612303)

[12.1 Study Design/Endpoints 23](#_Toc145612304)

[12.2 Sample Size/Accrual Rate 23](#_Toc145612305)

[12.3 Stratification Factors 24](#_Toc145612306)

[12.4 Analysis of Endpoints 24](#_Toc145612307)

[12.4.1 Analysis of Primary Endpoints 24](#_Toc145612308)

[12.4.2 Analysis of Secondary Endpoints 24](#_Toc145612309)

[12.5 Interim Analysis 24](#_Toc145612310)

[12.6 Reporting and Exclusions 24](#_Toc145612311)

[12.6.1 Evaluation of Toxicity 24](#_Toc145612312)

[12.6.2 Evaluation of Response 24](#_Toc145612313)

[13. Adverse Event Reporting Requirements 24](#_Toc145612314)

[13.1 Adverse Event Definition 25](#_Toc145612315)

[13.1.1 Investigational Agent or Device Risks (Expected Adverse Events) 25](#_Toc145612316)

[13.1.2 Adverse Event Characteristics and Related Attributions 25](#_Toc145612317)

[13.1.3 Recording of Adverse Events 25](#_Toc145612318)

[13.1.4 Reporting of AE to WCM IRB 25](#_Toc145612319)

[13.1.5 Reporting Events to Participants 26](#_Toc145612320)

[13.1.6 Events of Special Interest 26](#_Toc145612321)

[13.1.7 Reporting of Pregnancy 26](#_Toc145612322)

[13.2 Definition of SAE 26](#_Toc145612323)

[13.2.1 Reporting of SAE to IRB 26](#_Toc145612324)

[13.2.2 Reporting of SAE to FDA 26](#_Toc145612325)

[13.2.3 Reporting of SAE to <*Insert Pharmaceutical Company Name>* 27](#_Toc145612326)

[13.3 AE/SAE Follow Up 28](#_Toc145612327)

[13.4 Time Period and Frequency for Event Assessment and Follow Up 28](#_Toc145612328)

[14. Unanticipated Problems Involving Risks to Subjects or Others 29](#_Toc145612329)

[14.1 Definition of Unanticipated Problems Involving Risks to Subjects or Others (UPIRTSO) 29](#_Toc145612330)

[14.1.2 Unanticipated Problem Reporting 30](#_Toc145612331)

[15. Data and Safety Monitoring Plan (DSMP) 31](#_Toc145612332)

[16. References 33](#_Toc145612333)

[Appendix A 34](#_Toc145612334)

**Statement of Compliance**

1. [The trial will be carried out in accordance with International Conference on Harmonization Good Clinical Practice (ICH GCP) and the following:

- United States (US) Code of Federal Regulations (CFR) applicable to clinical studies (45 CFR Part 46, 21 CFR Part 50, 21 CFR Part 56, 21 CFR Part 312, and/or 21 CFR Part 812)

National Institutes of Health (NIH)-funded investigators and clinical trial site staff who are responsible for the conduct, management, or oversight of NIH-funded clinical trials have completed Human Subjects Protection and ICH GCP Training.

The protocol, informed consent form(s), recruitment materials, and all participant materials will be submitted to the Institutional Review Board (IRB) for review and approval. Approval of both the protocol and the consent form must be obtained before any participant is enrolled. Any amendment to the protocol will require review and approval by the IRB before the changes are implemented to the study. In addition, all changes to the consent form will be IRB-approved; a determination will be made regarding whether a new consent needs to be obtained from participants who provided consent, using a previously approved consent form.

# Confidentiality Statement

This document is confidential and is to be distributed for review only to investigators, potential investigators, consultants, study staff, and applicable independent ethics committees or institutional review boards. The contents of this document shall not be disclosed to others without written authorization from WCM, unless disclosure on ClinicalTrials.gov is federally required.

***For multi-site IIT only. Remove signature lines if single site study.***

**Weill Cornell Medicine**

**_________________________________________________________________**

**Institution Name**

**Erica Phillips**

**____________________________ _____________________________ 10.6.2023**

**Principal Investigator’s Name Principal Investigator’s Signature Date**

# List of Abbreviations

| **AE** | Adverse Event |
| --- | --- |
| **CFR** | Code of Federal Regulations |
| **CRF** | Case Report Form |
| **CTSC** | Clinical Translational Science Center |
| **DSMB** | Data Safety Monitoring Board |
| **DSMP** | Data Safety Monitoring Plan |
| **FDA** | Food and Drug Administration |
| **GCP** | Good Clinical Practice |
| **HIPAA** | Health Insurance Portability and Accountability Act of 1996 |
| **HRBFA** | Human Research Billing Analysis Form |
| **HUD** | Humanitarian Use Device |
| **ICF** | Informed Consent Form |
| **IDE** | Investigational Device Exemption |
| **IND** | Investigational New Drug |
| **IRB** | Institutional Review Board |
| **PHI** | Protected Health Information |
| **PI** | Principal Investigator |
| **REDCap** | Research Electronic Data Capture |
| **SAE**  **SNM** | Serious Adverse Event  Social Network Member |
| **SUSAR** | Suspected Unexpected Serious Adverse Reaction |
| **UIRTSO** | Unanticipated Problem Involving Risks to Subjects or Others |
| **WCM** | Weill Cornell Medicine |

# 1. Protocol Summary

Full Title: Reducing OBesity Using Social Ties (ROBUST): Randomized Controlled Trial Short Title: ROBUST- RCT

Clinical Phase: III

Principal Investigator: Erica Phillips, MD, MS

**Study Description:** Randomized controlled trial comparing two behavioral weight-loss interventions; a social-network enhanced lifestyle intervention (Intervention group) and an individual level lifestyle intervention (Control group), among Black and Hispanic adults with obesity over the course of 24 weeks. This study aims to test the feasibility and acceptability reforming interpersonal barriers and addressing social network barriers to behavior change, by engaging social network members in a lifestyle intervention for weight loss compared to the lifestyle intervention alone.

**Sample Size:** *N=* *66 (Intervention index participants)*

*N=132 (Intervention Social Network members)*

*N= 66 (Control participants)*

*N=264 (Total Sample Size)*

**Enrollment:** In the intervention group, this study will enroll 66 index participants and 132 social network member subjects. In the control group, this study will enroll up to 66 index participants.

**Study Population:** Individuals who self-identify as Non-Hispanic Black or Hispanic adults 18 years of age or older with a calculated BMI ≥ 30 kg/m2

**Enrollment Period:** *24 weeks*

**Study Design:** Randomized controlled trial comparing a social-network enhanced lifestyle (intervention group) to an individual-level lifestyle intervention (control group). Subjects in both arms (n=66 each) will receive the same number of behavioral coaching sessions (15 total) with four sessions conducted in person (baseline, week 8, week 16, and week 24), and the remaining 11 sessions conducted virtually. Subjects randomized to the social network intervention will nominate up to a maximum of two members of their social network (n=132) who will also be consented at baseline, complete a brief baseline interview, and attend the virtual coaching sessions with the randomized subject at weeks 3, 5, and 15. The social network members will also complete a close-out study interview at week 24.

**Description of Sites/**

**Facilities Enrolling**

**Participants:** Four sites including: Weill Cornell Internal Medicine Associates, Weill Cornell Urology locations in Manhattan & Brooklyn, and Weill Cornell Primary Care at Lower Manhattan. The study is not intended to include sites outside of the United States.

**Study Duration:** *3 years*

**Participant Duration:** 24 weeks (6 months)

**Study Agent/Device Name**

**Intervention Description:** The social-network enhanced lifestyle Intervention will target:

1. The relationship between participants and their social network members to induce a positive affect, social support, and collaborative problem solving (communal coping) through three communications skills training sessions.
2. Acquisition of behavioral skills that will enhance adherence to dietary and physical activity goals through one-on-one lifestyle coaching and dietary counseling sessions

**Primary Objectives:** Evaluate the feasibility of each participant in the ROBUST intervention engaging up to two personal social network members to participate in the study.

**Secondary Objectives:** Evaluate the impact of the ROBUST intervention on modifying the target social network barriers of: social undermining and unhealthy weight norms by activating a communal coping process within the personal network, and interpersonal conflicts by inducing positive affect to build resiliency.

Examine if positive changes in these social network processes result in greater adoption of healthier diets, increased physical activity, and weight loss at 24 weeks.

**Exploratory Objectives:** Examine the ROBUST Intervention’s effect on weight change and

related behaviors among network members who participate in the study.

Identify social network phenotypes that may be predictive of differential weight loss patterns.

**Primary Endpoints:** At least 75% attendance rate for the participant and their network members in their respective behavioral coaching sessions.

80% of participants or more to have at least one network member engage in the study.

85% of participants and their network members or more to complete the final study assessment.

**Secondary Endpoints:** Increase in positive communication and problem solving scores on the McMaster Family Assessment Device (FAD) for the participant and their social network members.

Decrease in social norms that promote unhealthy eating and physical inactivity (unhealthy weight norms).

Increase in participant physical activity and decrease in caloric intake

## 1.1 Schema


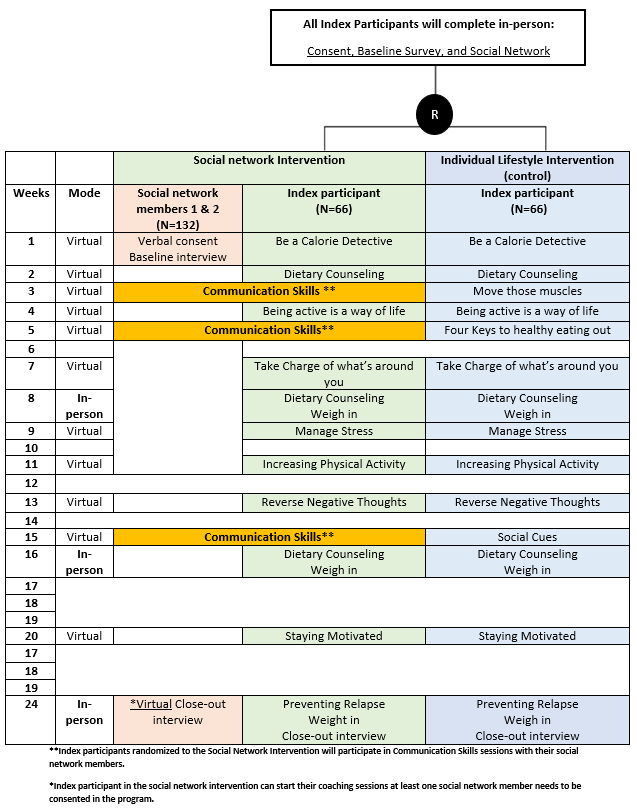


## 1.2 Study Objectives and End Points

### **1.2.1 Primary Objectives**

To evaluate the feasibility of each participant in the ROBUST intervention engaging up to two personal social network members to participate in the study.

### **1.2.2 Secondary Objectives**

To evaluate the impact of the ROBUST intervention on modifying the target social network barriers of social undermining and unhealthy weight norms by activating a communal coping process within the personal network, and interpersonal conflicts by inducing positive affect to build resiliency.

To examine if positive changes in these social network processes result in greater adoption of healthier diets, increased physical activity, and weight loss at 24 weeks.

### **1.2.3 Exploratory Objectives**

### To examine the ROBUST Intervention’s effect on weight change and related behaviors among social network members who participate in the study.

To identify social network phenotypes that may be predictive of differential weight loss patterns.

### **1.2.4 Primary Endpoints**

At least 75% attendance rate for the participant and their network members in their respective behavioral coaching sessions.

80% of participants or more to have at least one network member engage in the study.

85% of participants and their network members or more to complete the final study assessment.

### **1.2.5 Secondary Endpoints**

Increase in positive communication and problem solving scores on the McMaster Family Assessment Device (FAD) for the participant and their social network members.

Decrease in social norms that promote unhealthy eating and physical inactivity (unhealthy weight norms).

Increase in participant physical activity, and decrease in caloric intake.

# 2. Background

## 2.1 Scientific background of the problem

Eliminating the disproportionate burden of metabolic diseases has remained a pervasive objective for the United States healthcare system. It has become increasingly recognized that obesity is a constant factor of multiple chronic comorbidities, such as cardiovascular disease, Type 2-Diabetes (T2D), and certain forms of cancer among thousands of Americans. The Centers for Disease Control and Prevention (CDC) defines obesity as a “public health problem”.^1^ A study in New York city looking at the disproportionate rate differences in the prevalence of obesity among racial and ethnic groups highlights that racial minorities such as non-Hispanic Blacks (36.9%) and Hispanics (36.8%) are two times to have obesity and be subjected to other metabolic diseases then Asian/Pacific Islanders (16.5%) and 1.5 times more than non-Hispanic whites (27.9%).^2-4^

However, the explosive rise in the prevalence of obesity cannot be solely explained by individual factors alone. Researchers have recognized that social-environmental factors also contribute to obesity risk and inequities. Social influences in particular have a pervasive impact on obesity and related behaviors but are typically not addressed in interventions.^5,6-10^ Pilot studies focused on reducing negative social influences have been found to improve intervention effectiveness, address the obesity epidemic, and promote healthy behavior change by incorporating social network intervention strategies.^5,7,11^ This raises a critical need to develop efficacious and tailored approaches to reduce the prevalence of obesity across high-risk communities by addressing social influences on weight loss habits*.* ^9^

## 2.2 Behavioral Intervention to be tested

An innovative way to target social influence as a path to reduce the prevalence of obesity is through Social Network Interventions.^12,13, 23,24^ Social Network interventions are an intentional effort to modify or use the characteristics of social networks to improve, generate, and maintain healthy behaviors among individuals and populations. Researchers have leveraged social network intervention strategies, such as social support and social learning, to enact behavioral change related to drug use, weight-loss, and type 2 diabetes.^14-17^ Similarly, recent literature illustrated that social network characteristics, such as size, closeness, and density, can also influence a person’s health by shaping the access to information, opportunities, and social norms, thereby enabling or constraining behaviors.^18,19-22^ However, there have been a limited number of studies that have directly addressed or modified social network processes, beyond individual perceptions of social phenomena, to comprehensively address emergent social network influences that have been documented to be important to weight-loss and health behavior change. Hence, this proposed study will focus on implementing a behavioral social-network intervention to promote changes in weight loss behaviors. This study will target key social network processes while also prospectively characterizing the composition, structure, and functions of the networks over time.

## 2.3 Rationale

There are a limited number of studies that directly address or modify social network processes, to comprehensively address emergent social network influences that have been documented to be important to weight-loss and health behavior change. Hence, this pilot randomized controlled trial aims to use evidence-based approaches to promote healthy behavioral changes towards weight-loss, which includes addressing key network level processes among participants and their social network members through individualized coaching sessions, and personalized behavioral recommendations.

## 2.4 Risk/Benefit Assessment

### **2.4.1 Known Potential Risks**

Known potential risks of this study includes the following:

- Discomfort with personal information being discussed
- There may be other potential risks other than listed that we cannot predict

### **2.4.2 Known Potential Benefits**

Known potential benefits of this study include the following:

- Potential weight loss
- Gain knowledge of weight loss techniques and positive lifestyle changes
- Improvements to general health due to improved eating and physical activity habits

### **2.4.3 Assessment of Potential Risks and Benefits**

Participants will disclose personal information about lifestyle habits in communication skills sessions with study staff and a social network member. The participant may feel uncomfortable disclosing this information in front of other individuals. To minimize the risk of discomfort, study staff will limit the information discussed during the session and ensure that the participant verbally and physically confirms (consent) that they are comfortable discussing personal information.

## 2.5 Correlative Studies Background

**Not applicable**

# 3. Study Design

## 3.1 Overall Design

This randomized controlled trial comparing a social-network enhanced lifestyle intervention (intervention group) to an individual-level lifestyle intervention (control group) will be conducted among 132 adult individuals who self-identify as Black or Hispanic with obesity (BMI ≥ 30 kg/m2). The hypothesis being tested is that the multi-component social-network enhanced lifestyle intervention will be feasible, acceptable, and lead to positive change in key social network processes (communal coping, social norms, and affect) that will reduce established interpersonal barriers (social undermining, unhealthy social norms, and interpersonal conflict) to weight-loss. 132 index participants will be recruited and evenly randomized to either the social network intervention group or the control group. Both groups will be followed over 24 weeks (6 months), completing virtual coaching sessions on zoom, along with three in person dietary assessments and weigh-ins. Individuals randomized to the intervention will nominate up to two personal social network members who will participate with the index participants in three virtual communication skills sessions.


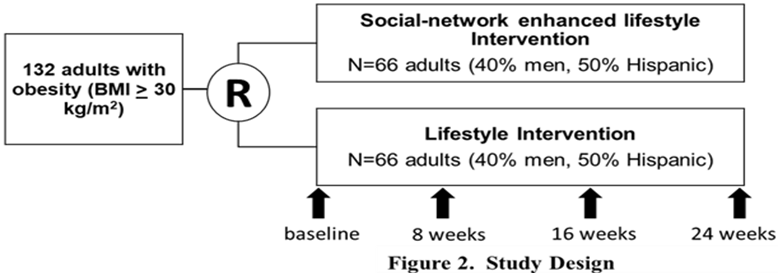


##

## 3.2 Scientific Rationale for Study Design

Our study is informed by the social cognitive theory (SCT), which posits that individual-level behaviors are shaped by a dynamic interaction between people (personal factors), behavior, and environments. This interaction is demonstrated by the construct called Reciprocal Determinism. Personal factors, environmental factors, and behavior continuously interact through influencing and being influenced by each other. **An intervention must target all three elements to invoke lasting change in behaviors**. The ROBUST intervention will specifically target the: 1) participants' self-efficacy and affective state by teaching them (and their participating network members) how to induce a positive affect and affirmative state using a simple, teachable self-directing script; 2) acquisition of behavioral skills in self-regulation that will enhance adherence to dietary and physical activity goals; and 3) environmental factors of positive reinforcement, social support, weight-related norms and collaborative problem solving (communal coping) by implementing virtual one-on-one lifestyle coaching and communication skills training within the personal network. By intervening and measuring key variables at the individual and interpersonal level, we have married the SCT to Social Network Theory (SNT). SNT elucidates the role of social relationships and social structure in transmitting information, channeling interpersonal influence, and enabling attitudinal or behavioral change.

## 3.3 Justification for Dose

Using the Guidelines (2013) for Managing Overweight and Obesity in Adults as a framework, the number of sessions selected for the intervention and control group is consistent with the principal components of comprehensive lifestyle modification, which include diet, physical activity, and behavior therapy. To lose weight, the guidelines recommend participation for 6 months in high-intensity programs that provide 14 or more counseling sessions with a trained interventionist.^25,26^

## 3.4 End of Study Definition

A participant is considered to have completed the study if he or she has completed all phases of the study including the last visit. The end of the study is defined as completion of the last visit or the completion of the withdrawal procedure.

# 4. Subject Selection

## 4.1 Study Population

Subjects that meet the inclusion criteria will be eligible for participation in this study.

Social network subjects identified by index participants that are willing to be engaged in the study will also be eligible for participation in this study.

## 4.2 Inclusion Criteria Index Participant

1. Black race or Hispanic ethnicity

2. Male or Female 18 years of age or older

3. Calculated BMI > 30kg/m^2^ from objectively measured height and weight by study staff

4. Access & willingness to use program food intake app via home computer or a smartphone

5. Ability to identify at least one adult social network member who will participate in the study

## 4.3 Exclusion Criteria Index Participant

1. Active enrollment in a weight-loss program, use of weight-loss medications, or planning weight-loss surgery

2. Advanced medical illness, dementia, hospitalization, injury, or pregnancy that inhibits regular physical activity

3. Contraindications to exercise based on the Physical Activity Readiness Questionnaire or lack of clearance from a health care provider

4. Unresolved Food insecurity

5. Speaks a language other than English or Spanish

## 4.4 Inclusion Criteria Social Network Member

1. Male or Female 18 years of age or older
2. Access to the internet or a smartphone

## 4.5 Exclusion Criteria Social Network Member

1. Speaks a language other than English or Spanish

## 4.6 Lifestyle Considerations

**Not applicable**

## 4.7 Screen Failures

Screen failures are defined as participants who consent to participate in the clinical trial but are not subsequently randomly assigned to the study intervention or entered in the study. A minimal set of screen failure information is required to ensure transparent reporting of screen failure participants, to meet the Consolidated Standards of Reporting Trials (CONSORT) publishing requirements and to respond to queries from regulatory authorities. Minimal information includes demography, screen failure details, eligibility criteria, and any serious adverse event (SAE). Individuals who do not meet the criteria for participation in this trial (screen failure) because of an active enrollment in a weight loss program, food insecurity, or lack of clearance from a medical provider may be rescreened. Rescreened participants should be assigned the same participant number as for the initial screening.

## 4.6 Strategies for Recruitment and Retention

Recruitment Strategies:

- An estimated 800 Black or Hispanic individuals 18 years of age or older will need to be screened in order to reach the target enrollment of 132 participants.
- The anticipated accrual rate is 8 participants per month (6% of target enrollment)
- Recruitment will take place at four outpatient clinic sites Weill Cornell Internal Medicine Associates, Weill Cornell Urology @ Brooklyn, Weill Cornell Urology @ Upper East Side, and Weill Cornell Primary Care at Lower Manhattan.
- Potential participants will be identified through a TRAC request in EPIC specific to those clinical sites. Study staff will contact the medical provider to verify that the participant is fit to potentially participate in the study and ask their permission to contact the potential participant. Participants will be contacted via phone call, email or regular mail based on their indicated preferences for communication in the electronic health record.
- Recruitment strategies will include mailing potential participants flyers describing the study with the study team contact information.

Retention Strategies:

- - Collection of monthly screening, enrollment, and retention logs
  - Index participants will be compensated $25 for each in person visit
  - Social network participants will be compensated $25 for each completed survey
  - Participants will receive text and call reminders for each scheduled visit

# 5. Registration Procedures

## 5.1 Subject Registration (WCM only)

Subjects will be registered within the WRG-CT as per the standard operating procedure for Subject Registration.

## 5.2 Subject Registration (Sub-sites)

**Not applicable**

# 6. Study Procedures

## 6.1 Schedule of Assessments

Table 1. Schedule of trial events

| **Study Measures and Assessment Schedule Week #** | | | |
| --- | --- | --- | --- |
| **Construct** | **Measure** | **Index** | **SN** |
| **Personal** | - Socio-demographic and biology: age, sex, race/ethnicity, marital status, education, employment, occupation, medical conditions, medications, food security  - Objective anthropometrics: height, weight, BMI, waist to hip ratio, waist circumference  - Self-reported height and weight *(participating social network only)*  - Self-efficacy: 9 item self-efficacy scale  - Perceived Stress: Cohen's perceived stress scale 3-item  - Positive Affect: The Positive and Negative Affect Schedule (PANAS) | 0  0, 8, 16, 24  0, 24  0, 24  0, 24 | 0  0, 24  0, 24  0, 24  0, 24 |
| **Environmental** | - Support and Undermining: Sallis Social Support and Eating Habits/ Exercise  - Health norms: Weight Related Social Norms Scale  - Social network interview: structure, composition, and functions (interpersonal conflict, undermining, communication, support, obesity-related norms)  - Communal Coping: verbal encouragement and collaborative problem solving | 0, 24  0, 24  0, 24  0,8,16,24 | 0, 24 |
| **Behavioral** | - Self-reported physical activity: Paffenbarger Physical Activity Questionnaire  - Objectively measured physical activity: Fitbit fitness tracker  - Dietary intake: three-day food record | 0, 8, 16, 24  0, 8, 16, 24  0, 8, 16, 24 | 0, 24  0, 24 |

### **6.1.1 Screening Visit (*14 days before start of treatment*)**

- Informed consent

### **6.1.2 Treatment Phase**

Eligible participants will be randomly assigned to the social network intervention or control groups in a 1:1 ratio using a numerical randomization scheme developed by the statistical team. Following randomization, participants that are randomized to the social network intervention group will complete 3 in person communication skills sessions with their social network members during weeks 3, 5, and 15. Instead of completing communication skills sessions, participants randomized to the control group will complete regular virtual lifestyle coaching sessions with the study team that consists of the following topics: Keep your health healthy (week 3), Eat well away from home (week 5), and Social Cues (Week 15). All other sessions will focus on the topics below and will be the same across both treatment groups.

Note: The weeks that are not listed in the treatment phase below are weeks that will be treatment breaks.

### **Visit 1 (Intake; Week 0)**

### Review accuracy of completed baseline survey (10 minutes)

### Conduct anthropometrics (15 minutes)

### Conduct Social Network Survey (15 minutes)

### Set up Program Equipment & set coaching schedule (45 minutes)

### **Visit 2 (Track your food and physical activity; Week 1)**

- - - - 1. Check in (5 minutes)
        2. Review self-regulations skills (10 minutes)
        3. Review workbook lesson (30 minutes)
        4. Homework & set new/or revise SMART Goal (5 minutes)

### **Visit 3 (Dietary Counseling, Week 2)**

- - - - 1. Review of 3 day food record (10 minutes) Activities (40 minutes)
        2. Review workbook lesson (30 minutes)
        3. Homework & set new/or revise dietary SMART Goal (5 minutes)

### **Visit 4 (Communication Skills or Keep Your Heart Healthy, Week 3)**

### Check in (5 minutes)

### Review self-regulations skills (10 minutes)

### Review workbook lesson (30 minutes)

### Homework & set new/or revise SMART Goal (5 minutes)

### **Visit 5 (Find Time for Fitness and Get Active, Week 4)**

### Check in (5 minutes)

### Review self-regulations skills (10 minutes)

### Review workbook lesson (30 minutes)

### Homework & set new/or revise SMART Goal (5 minutes)

### **Visit 6 (Communication Skills or Eat Well Away from Home, Week 5)**

- - - - 1. Check in (5 minutes)
        2. Review self-regulations skills (10 minutes)
        3. Review workbook lesson (30 minutes)
        4. Homework & set new/or revise SMART Goal (5 minutes)

### **Visit 7 (Burn more calories that you can take it, Week 7)**

### Check in (5 minutes)

### Review self-regulations skills (10 minutes)

### Review workbook lesson (30 minutes)

### Homework & set new/or revise SMART Goal (5 minutes)

### **Visit 8 (Dietary Counseling, Week 8)**

### Review of 3 day food record (10 minutes)

### Review workbook lesson (30 minutes)

### Homework & set new/or revise dietary SMART Goal (5 minutes)

### **Visit 9 (Manage Stress, Week 9)**

- - - - 1. Check in (5 minutes)
        2. Review self-regulations skills (10 minutes)
        3. Review workbook lesson (30 minutes)
        4. Homework & set new/or revise SMART Goal (5 minutes)

### **Visit 10 (Get more active, Week 11)**

- - - - 1. Check in (5 minutes)
        2. Review self-regulations skills (10 minutes)
        3. Review workbook lesson (30 minutes)
        4. Homework & set new/or revise SMART Goal (5 minutes)

### **Visit 11 (Take charge of your thoughts, Week 13)**

- - - - 1. Check in (5 minutes)
        2. Review self-regulations skills (10 minutes)
        3. Review workbook lesson (30 minutes)
        4. Homework & set new/or revise SMART Goal (5 minutes)

### **Visit 12 (Communication Skills or Social Cues, Week 15)**

- - - - 1. Check in (5 minutes)
        2. Review self-regulations skills (10 minutes)
        3. Review workbook lesson (30 minutes)
        4. Homework & set new/or revise SMART Goal (5 minutes)

### **Visit 13 (Dietary Counseling, Week 16)**

- - - - 1. Review of 3 day food record (10 minutes)
        2. Review workbook lesson (30 minutes)
        3. Homework & set new/or revise dietary SMART Goal (5 minutes)

### **Visit 14 (Staying Motivated, Week 20)**

- - - - 1. Check in (5 minutes)
        2. Review self-regulations skills (10 minutes)
        3. Review workbook lesson (30 minutes)
        4. Homework & set new/or revise SMART Goal (5 minutes)

### **Follow-up Phase**

### **Visit 15 (Close out; Week 24)**

### Close out survey for control group, and social network intervention group and their social network members

### Preventing relapse nutrition session for the social network intervention group and the control group

# 7. Study Intervention

##

## 7.1 Study Intervention Description

The study intervention is a social network intervention which consists of the involvement of participants social network members. Study participants will complete 3 communication skills sessions with their social network members which will involve the creation and utilization of positive affirmations and strategies that social network members will use to reduce undermining participants during their weight loss journey.

- Communication Skills session outline: See Appendix

## 7.2 Availability

The intervention is an investigational agent that uses scripts and worksheets that are created by the study team. The intervention does not require any supplies from Pharmaceutical or Device Companies.

## 7.3 Acquisition and Accountability

Retention log – The study staff will maintain a record of participants who complete the social network intervention.

## 7.4 Formulation, Appearance, Packaging, and Labeling

Please see appendix for worksheets.

## 7.5 Product Storage and Stability

**Not Applicable**

## 7.6 Preparation

**Not applicable**

## 7.7 Dosing and Administration

Participants receiving the social network intervention will complete communication skills sessions during weeks 3, 5, and 15 of the study. Participants in the control group will complete regular discussion sessions during week 3,5, and 15 of the study which will consist of the following topics: Move those muscles, Keys to health eating out, and social cues, respectively. Those who continue in the study will only receive each session one time.

### **7.7.1 Dosing Delays/Dose Modifications**

**Not applicable**

## 7.8 General Concomitant Medication and Supportive Care Guidelines

**Not applicable**

## 7.9 Duration of Therapy and Criteria for Removal from Study

All control and treatment group participants will receive the study intervention for 24 weeks. A subject’s follow-up in the study will end after one of the following applies:

- Subject’s voluntary withdrawal
- Subject lost to follow-up
- Adverse medical event in which the participant has been advised to halt all physical activity or needs to initiate a new medically prescribed diet
- Subject death
- Completion of all scheduled study follow-up appointments

## 7.10 Duration of Follow Up

Subjects will be followed for 6 month after removal from study or until death, whichever occurs first. Subjects removed from study for unacceptable adverse events will be followed until resolution or stabilization of the adverse event.

## 7.11 Measures to Minimize Bias: Randomization and Blinding

Index participants will be randomized after completion of the social network interview either to the ROBUST intervention or lifestyle intervention, using a random number generator. All index participants, regardless of randomization group, will complete a social network interview to characterize their personal network prior to randomization. The same social network interview will be conducted at 24 weeks. All participants will also receive the same number of coaching sessions, as shown in **Table 1**. They will attend four sessions in person (baseline, week 8, week 16, and week 24), and the remaining 11 sessions will be conducted as a virtual visit. The in-person visits will occur at the Weill Cornell Clinical and Translational Science Center's dedicated research facility. During the in-person sessions, the participant will receive 1:1 dietary counseling with a registered dietician who has been trained on the study protocol. The study research staff complete anthropometric measurements (height, weight, and body composition). Participants randomized to the intervention group will be expected to attend virtual visits at weeks 3, 5, and 15 with their social network member.

Consenting of study participants as well as all study assessments (i.e. baseline, 8 ,16 & 24 weeks) will be conducted by an assessor who is not responsible or involved in the delivery of the coaching sessions, dietary counseling or communication skills sessions. While the study PI & co-investigators will be blinded, the same will not be possible for the trained coach delivering the intervention. Due to budget limitations, having two coaches (i.e. one for the control group and for the intervention group) is not feasible. Since the communication skills training sessions are designed to occur between the index participant and consented social network members, the implication of contamination is reduced as control group participants will not have any consented social network members participating in the study.

*.*

## 7.12 Study Intervention/Follow-up Compliance

Adherence to the protocol will be assessed and verified through the completion of REDCap forms each time that a study team member speaks with participants. This REDCap form will contain questions to verify the date of each coaching session and verify the collection of any related materials (i.e. verify collection of food logs, etc). It is mandatory for study team members to complete the REDCap form and the phone log form as these documents will be used to calculate study intervention compliance.

5 attempts will be made to get participants to return for study follow up appointments via text and calls to the participant. A participant will be considered “lost to follow up” when 3 consecutive sessions have been missed and the study team can no longer get in contact with the participant. Participants that are not lost to follow up will be considered as no longer participating in the study when week 24 of the study (close out interview) has been completed.

# 8. Study Intervention Discontinuation and Participant Discontinuation/Withdrawal

## 8.1 Discontinuation of Study Intervention

Criteria for discontinuing the study intervention is as follows:

- Cardiovascular, pulmonary, hypoglycemia: Adverse events will be defined as cardiovascular (MI, CVA, death), acute shortness of breath, or hypoglycemia precipitated by changes in dietary intake or the physical activity chosen for the study requiring hospitalization/emergency room visit or a physician office visit. Non-routine office visits for cardiac or pulmonary disease or diabetes are expected and not serious unless they lead to the above events.
- Major depressive episode or anxiety attack: Infrequent likelihood. A participant may become depressed, anxious or severely embarrassed if friends/family/acquaintances find out that the person is participating in and/or not losing weight in the program. Patients have the option of not answering any questions they do not want to. As this is a voluntary study, a participant is free to withdraw from the study at any point if they feel uncomfortable or do not want to continue.
- Medication Side Effects: Infrequent risks. The study does not prescribe medication. Weight loss can cause a change in the dose of medication needed to treat common health conditions such as hypertension, diabetes and thyroid dysfunction. Participants will be required to communicate their participation in the study along with their weight loss to a primary care physician.
- Musculoskeletal: Musculoskeletal injury due to the physical activity chosen for this study resulting in hospitalizations, emergency department visits, or treatment for fracture in any setting will be considered expected serious adverse events. Non-routine office visits for musculoskeletal injury are expected and not serious unless lead to the above events. Temporary minor incapacitation due to muscle soreness is also expected and not serious.

Discontinuation from the social network intervention does not mean discontinuation from the study, and remaining study procedures should be completed as indicated by the study protocol. If a clinically significant finding is identified (including, but not limited to changes from baseline) after enrollment, the investigator or qualified designee will determine if any change in participant management is needed. Any new clinically relevant finding will be reported as an adverse event (AE).

The data to be collected at the time of study intervention discontinuation will include the following:

- Adverse event form
- If applicable, withdrawal form

## 8.2 Participant Discontinuation/Withdrawal from the Study

Participants are free to withdraw from participation in the study at any time upon request.

An investigator may discontinue or withdraw a participant from the study for the following reasons:

- Pregnancy
- Significant study intervention non-compliance
- If any clinical adverse event (AE), laboratory abnormality, or other medical condition or situation occurs such that continued participation in the study would not be in the best interest of the participant
- Disease progression which requires discontinuation of the study intervention
- If the participant meets an exclusion criterion (either newly developed or not previously recognized) that precludes further study participation
- Participant lost to follow-up after several attempts to contact subject to schedule study visit.

The reason for participant discontinuation or withdrawal from the study will be recorded on the Withdrawal Case Report Form (CRF). Subjects who sign the informed consent form and are randomized but do not receive the study intervention will be replaced. Subjects who sign the informed consent form, and are randomized and receive the study intervention, and subsequently withdraw, or are withdrawn or discontinued from the study, will not be replaced.

## 8.3 Lost to Follow Up

A participant will be considered lost to follow-up if he or she fails to return for 3 scheduled visits and is unable to be contacted by the study site staff.

The following actions must be taken if a participant fails to return to the clinic for a required study visit:

- The site will attempt to contact the participant and reschedule the missed visit within 2 days and counsel the participant on the importance of maintaining the assigned visit schedule and ascertain if the participant wishes to and/or should continue in the study.
- Before a participant is deemed lost to follow-up, the investigator or designee will make every effort to regain contact with the participant (where possible, 3 telephone calls and, if necessary, a certified letter to the participant’s last known mailing address or local equivalent methods). These contact attempts should be documented in the participant’s medical record or study file.
- Should the participant continue to be unreachable, he or she will be considered to have withdrawn from the study with a primary reason of lost to follow-up.

# 9. Correlative/Special Studies

**Not applicable**

## 9.1 Laboratory Correlative Studies

**Not applicable**

**9.1.1 Title – Laboratory Correlative Study #1 - Not applicable**

**9.1.1.1 Collection of Specimen(s) - Not applicable**

**9.1.1.2 Handling of Specimen(s) - Not applicable**

**9.1.1.3 Shipping of Specimen(s) (if multicenter) - Not applicable**

**9.1.1.4 Site(s) Performing Correlative Study (if multicenter) - Not applicable**

## 9.2 Special Studies

**Not applicable**

**9.2.1 Title – Special Correlative Study #1 - Not applicable**

**9.2.1.1 Assessment - Not applicable**

**9.2.1.2 Method of Assessment - Not applicable**

**9.2.1.3 Timing of Assessment - Not applicable**

# 10. Measurement of Effect

## 10.1 Response Criteria

To assess the primary objective, feasibility will be assessed based on rates of recruitment, study conduct (intervention and enactment fidelity), retention and acceptability of the study. Descriptive statistics will be used to summarize recruitment rates. The screening-to-enrollment (STE) ratio will be defined and calculated as the proportion of people who consented to those for whom screening was attempted. Adherence to several aspects (session attendance, dietary, physical activity and self-weights) of the intervention will be tracked to calculate total counts of increase or decrease in adherence. Treatment fidelity will be summarized as an average of the proportion of treatment elements delivered correctly.

To assess the secondary objective, we will examine summary statistics, histograms, and scatter plots of the measured covariates and outcomes for outliers and data trends.

## 10.2 Duration of Response

Duration of overall response: 24 weeks

Duration of stable disease: Stable disease is measured from the start of the treatment until the criteria for progression are met, taking as reference the smallest measurements recorded since the treatment started.

## 10.3 Progression-Free Survival

**Not Applicable**

## 10.4 Other Response Parameters

**Not applicable**

# 11. Data Reporting / Regulatory Considerations

## 11.1 Data Collection

The data collection plan for this study is to utilize REDCap to capture all treatment, toxicity, efficacy, and adverse event data for all enrolled subjects.

### **11.1.1 REDCap**

REDCap (Research Electronic Data Capture) is a free data management software system that is fully supported by the Weill-Cornell Medical Center CTSC. It is a tool for the creation of customized, secure data management systems that include Web-based data-entry forms, reporting tools, and a full array of security features including user and group based privileges, authentication using institution LDAP system, with a full audit trail of data manipulation and export procedures. REDCap is maintained on CTSC-owned servers that are backed up nightly and support encrypted (SSL-based) connections. Nationally, the software is developed, enhanced and supported through a multi-institutional consortium led by the Vanderbilt University CTSA.

## 11.2 Regulatory Considerations

### **11.2.1 Institutional Review Board/Ethics Committee Approval**

As required by local regulations, the Investigator will ensure all legal aspects are covered, and approval of the appropriate regulatory bodies obtained, before study initiation.

Before initiation of the study at each study center, the protocol, the ICF, other written material given to the patients, and any other relevant study documentation will be submitted to the appropriate Ethics Committee. Written approval of the study and all relevant study information must be obtained before the study center can be initiated or the IP is released to the Investigator. Any necessary extensions or renewals of IRB approval must be obtained for changes to the study, such as amendments to the protocol, the ICF, or other study documentation. The written approval of the IRB together with the approved ICF must be filed in the study files.

The Investigator will report promptly to the IRB any new information that may adversely affect the safety of the patients or the conduct of the study. The Investigator will submit written summaries of the study status to the IRB as required. On completion of the study, the IRB will be notified that the study has ended.

All agreed protocol amendments will be clearly recorded on a protocol amendment form and will be signed and dated by the original protocol approving signatories. All protocol amendments will be submitted to the relevant institutional IRB for approval before implementation, as required by local regulations. The only exception will be when the amendment is necessary to eliminate an immediate hazard to the trial participants. In this case, the necessary action will be taken first, with the relevant protocol amendment following shortly thereafter.

Once protocol amendments or consent form modifications are implemented at the lead site, Weill Cornell Medicine, updated documents will be provided to participating sites, as applicable. Weill Cornell Medicine must approve all consent form changes prior to local IRB submission.

Relevant study documentation will be submitted to the regulatory authorities of the participating countries, according to local/national requirements, for review and approval before the beginning of the study. On completion of the study, the regulatory authorities will be notified that the study has ended.

### **11.2.2 Ethical Conduct of the Study**

The Investigators and all parties involved should conduct this study in adherence to the ethical principles based on the Declaration of Helsinki, GCP, ICH guidelines and the applicable national and local laws and regulatory requirements.

This study will be conducted under a protocol reviewed and approved by the applicable ethics committees and investigations will be undertaken by scientifically and medically qualified persons, where the benefits of the study are in proportion to the risks.

### **11.2.3 Informed Consent**

The investigator or qualified designee must obtain documented consent according to ICH-GCP and local regulations, as applicable, from each potential subject or each subject’s legally authorized representative prior to participating in the research study. Subjects who agree to participate will sign the approved informed consent form and will be provided a copy of the signed document.

The initial ICF, any subsequent revised written ICF and any written information provided to the subject must approved by IRB prior to use. The ICF will adhere to IRB requirements, applicable laws and regulations.

### **11.2.4 Compliance with Trial Registration and Results Posting Requirements**

Under the terms of the Food and Drug Administration Modernization Act (FDAMA) and the Food and Drug Administration Amendments Act (FDAAA), the Sponsor-Investigator of the trial is solely responsible for determining whether the trial and its results are subject to the requirements for submission to <http://www.clinicaltrials.gov>. Information posted will allow subjects to identify potentially appropriate trials for their disease conditions and pursue participation by calling a central contact number for further information on appropriate trial locations and trial site contact information.

### **11.2.5 Record Retention**

Essential documents are those documents that individually and collectively permit evaluation of the study and quality of the data produced.  After completion of the study, all documents and data relating to the study will be kept in an orderly manner by the Investigator in a secure study file.  Essential documents should be retained for 2 years after the final marketing approval in an ICH region or for at least 2 years since the discontinuation of clinical development of the IP. In addition, all subjects medical records and other source documentation will be kept for the maximum time permitted by the hospital, institution, or medical practice. 

12. Statistical Considerations

## 12.1 Study Design/Endpoints

This randomized controlled trial will use the following primary endpoints as the promising range to justify further testing of this agent:

- At least 75% attendance rate for the participant and their network members in their respective behavioral coaching sessions.
- 80% of participants or more to have at least one network member engage in the study.
- 85% of participants and their network members or more to complete the final study assessment.

## 12.2 Sample Size/Accrual Rate

The anticipated accrual rate is 8 participants per month (6% of target enrollment).

## 12.3 Stratification Factors

**Not applicable**

## 12.4 Analysis of Endpoints

### **12.4.1 Analysis of Primary Endpoints**

Feasibility will be assessed based on rates of recruitment, study conduct (intervention and enactment fidelity), retention and acceptability of the study. Descriptive statistics will be used to summarize recruitment rates for clinical and faith-based organizations separately. The screening-to-enrollment (STE) ratio will be defined and calculated as the proportion of people who consented to those for whom screening was attempted. The reasons for declining participation will be grouped into categories and the proportion described.

Adherence to several aspects (session attendance, dietary, physical activity and self-weights) of the intervention will be tracked. The proportion of coaching sessions attended by the index participant and social network member across the 24 weeks will be calculated separately. Additionally, a tally of each self-regulation behavior (total # of days weighing, total # of days self-monitoring dietary intake, & total # of days wearing fitness tracker) will be summarized.

Treatment fidelity will be summarized as an average of the proportion of treatment elements delivered correctly. A correlation (Pearson) between the self-reported checklist and objectively measured checklist will be conducted. The retention rate will be defined as the proportion of participants who complete the 24-week assessment among those who completed the baseline assessment. Sample sizes and the proportion of missing data will be calculated for each measure collected as baseline, follow-up visits and study completion. Chi-Squared tests (or Fisher's exact tests) and two-independent-sample t-tests (or Wilcoxon rank-sum tests) will be used, as appropriate, to examine whether baseline characteristics predict retention and to identify differences in dropout rates between the intervention and control group. The qualitative satisfaction data will be content analyzed. Two study personnel will generate a priori codes corresponding to the interview guide (e.g., reactions to enrolling network members). One study personnel will apply the coding scheme to each interview summary, and then the two will formalize agreement or disagreement regarding the derived codes.

### **12.4.2 Analysis of Secondary Endpoints**

We will examine summary statistics, histograms, and scatter plots of the measured covariates and outcomes for outliers and data trends. Analyses of differences between those completing and not completing the study and the within-group changes during the intervention will be performed according to modified intention-to-treat principles. Multivariate linear regression (MLR) models will be used to examine the relationship between changes in the social network processes and exposure (the social network lifestyle intervention vs the lifestyle intervention), adjusted for covariates selected from univariate analyses; potential covariates for MLR are categorized by theoretical construct. We will also explore the effect of gender by including a three-way interaction (gender*social support*weight loss), although we recognize that we might not have enough power to detect such interactions.

To further explore the effect of the intervention, we will do sensitivity analysis: (1) Multivariable linear regression using weight loss as continuous outcome variables (a change in weight and percent weight change), adjusting for baseline covariates distributed differently between intervention groups; (3) test interaction terms between treatment group and other covariates; and (4) examine completers only.

## 12.5 Interim Analysis

**Not applicable**

## 12.6 Reporting and Exclusions

### **12.6.1 Evaluation of Toxicity**

Not applicable

### **12.6.2 Evaluation of Response**

All subjects included in the study will be assessed for response to treatment if they have received at least once coaching session after randomization given the intent-to-treat analysis that will be applied.

# 13. Adverse Event Reporting Requirements

Adverse event (AE) monitoring and reporting is a routine part of every clinical trial. The investigator will be required to provide appropriate information concerning any findings that suggest significant hazards, contraindications, side effects, or precautions pertinent to the safe use of the drug or device under investigation. Safety will be monitored by evaluation of adverse events reported by subjects or observed by investigators or research staff, as well as by other investigations such as clinical laboratory tests, x-rays, electrocardiographs, etc.

## 13.1 Adverse Event Definition

An adverse event (also referred to as an adverse experience) can be any unfavorable and unintended sign (e.g., an abnormal laboratory finding), symptom, or disease temporally associated with the use of a drug, and does not imply any judgment about causality. An adverse event can arise with any use of the drug (e.g., off-label use, use in combination with another drug) and with any route of administration, formulation, or dose, including an overdose.

### **13.1.1 Investigational Agent or Device Risks (Expected Adverse Events)**

Expected adverse events that could result from people receiving the intervention include:

Cardiovascular, pulmonary, hypoglycemia: Adverse events will be defined as cardiovascular (MI, CVA, death), acute shortness of breath, or hypoglycemia precipitated by changes in dietary intake or the physical activity chosen for the study requiring hospitalization/emergency room visit or a physician office visit. Non-routine office visits for cardiac or pulmonary disease or diabetes are expected and not serious unless they lead to the above events.

Major depressive episode or anxiety attack: Infrequent likelihood. A participant may become depressed, anxious or severely embarrassed if friends/family/acquaintances find out that the person is participating in and/or not losing weight in the program. Patients have the option of not answering any questions they do not want to. As this is a voluntary study, a participant is free to withdraw from the study at any point if they feel uncomfortable or do not want to continue.

Medication Side Effects: Infrequent risks. The study does not prescribe medication. Weight loss can cause a change in the dose of medication needed to treat common health conditions such as hypertension, diabetes and hypothyroidism. Participants will be required to communicate their participation in the study along with their weight loss to a primary care physician.

Musculoskeletal: Musculoskeletal injury due to the physical activity chosen for this study resulting in hospitalizations, emergency department visits, or treatment for fracture in any setting will be considered expected serious adverse events. Non-routine office visits for musculoskeletal injury are expected and not serious unless lead to the above events. Temporary minor incapacitation due to muscle soreness is also expected and not serious.

### **13.1.2 Adverse Event Characteristics and Related Attributions**

During the conduct of this study, we will use the WCM Data Safety Monitoring Committee Adverse Event reporting system.

**WCM grade:**

- **Attribution** of the AE:
  - Definite – The AE *is clearly related* to the study treatment.
  - Probable – The AE *is likely related* to the study treatment.
  - Possible – The AE *may be related* to the study treatment.
  - Unlikely – The AE *is doubtfully related* to the study treatment.
  - Unrelated – The AE *is clearly NOT related* to the study treatment.

### **13.1.3 Recording of Adverse Events**

All adverse events will be recorded on a subject specific AE log. The AE log will be maintained by the research staff and kept in the subject’s research chart.

### **13.1.4 Reporting of AE to WCM IRB**

All AEs occurring on this study will be reported to the IRB according to the IRB policy, which can be accessed via the following link: <http://researchintegrity.weill.cornell.edu/forms_and_policies/forms/Immediate_Reporting_Policy.pdf>.

### **13.1.5 Reporting Events to Participants**

**Not applicable**

### **13.1.6 Events of Special Interest**

**Not applicable**

### **13.1.7 Reporting of Pregnancy**

Participants must report any instance of pregnancy, in which they will be removed from the study.

## 13.2 Definition of SAE

SAEs include death, life threatening adverse experiences, hospitalization or prolongation of hospitalization, disability or incapacitation, overdose, congenital anomalies and any other serious events that may jeopardize the subject or require medical or surgical intervention to prevent one of the outcomes listed in this definition.

### **13.2.1 Reporting of SAE to IRB**

All SAEs occurring on this study will be reported to the IRB according to the IRB policy, which can be accessed via the following link:

<http://researchintegrity.weill.cornell.edu/forms_and_policies/forms/Immediate_Reporting_Policy.pdf>.

### **13.2.2 Reporting of SAE to FDA [For Protocols Where WCMC is the Sponsor-Investigator]**

**Not applicable**

### **13.2.3 Reporting of SAE to <*Insert Pharmaceutical Company Name>***

**Not applicable**

##

## 13.3 AE/SAE Follow Up

All SAEs and AEs reported during this study will be followed until resolution or until the investigator confirms that the AE/SAE has stabilized and no more follow-up is required. This requirement indicates that follow-up may be required for some events after the subject discontinues participation from the study.

## 13.4 Time Period and Frequency for Event Assessment and Follow Up

The occurrence of an adverse event (AE) or serious adverse event (SAE) may come to the attention of study personnel during study visits and interviews of a study participant presenting for medical care, or upon review by a study monitor.

All AEs including local and systemic reactions not meeting the criteria for SAEs will be captured on the appropriate case report form (CRF). Information to be collected includes event description, time of onset, clinician’s assessment of severity, relationship to study product (assessed only by those with the training and authority to make a diagnosis), and time of resolution/stabilization of the event. All AEs occurring while on study must be documented appropriately regardless of relationship. All AEs will be followed to adequate resolution.

Any medical condition that is present at the time that the participant is screened will be considered as baseline and not reported as an AE. However, if the study participant’s condition deteriorates at any time during the study, it will be recorded as an AE.

Changes in the severity of an AE will be documented to allow an assessment of the duration of the event at each level of severity to be performed. AEs characterized as intermittent require documentation of onset and duration of each episode.

Study team members will record all reportable events with start dates occurring any time after informed consent is obtained until 7 (for non-serious AEs) or 30 days (for SAEs) after the last day of study participation. At each study visit, the investigator will inquire about the occurrence of AE/SAEs since the last visit. Events will be followed for outcome information until resolution or stabilization.

**14. Unanticipated Problems Involving Risks to Subjects or Others**

## 14.1 Definition of Unanticipated Problems Involving Risks to Subjects or Others (UPIRTSO)

The Office for Human Research Protections (OHRP) considers unanticipated problems involving risks to participants or others to include, in general, any incident, experience, or outcome that meets **all** of the following criteria:

- Unexpected in terms of nature, severity, or frequency given (a) the research procedures that are described in the protocol-related documents, such as the Institutional Review Board (IRB)-approved research protocol and informed consent document; and (b) the characteristics of the participant population being studied;
- Related or possibly related to participation in the research (“possibly related” means there is a reasonable possibility that the incident, experience, or outcome may have been caused by the procedures involved in the research); and
- Suggests that the research places participants or others at a greater risk of harm (including physical, psychological, economic, or social harm) than was previously known or recognized.

### **14.1.2 Unanticipated Problem Reporting**

The investigator will report unanticipated problems (UPIRTSOs) to the reviewing Institutional Review Board (IRB) and to the Data Coordinating Center (DCC)/lead principal investigator (PI). Further details of the UPIRTSO report are included in the SOP. The UPIRTSO report will include the following information:

- Protocol identifying information: protocol title and number, PI’s name, and the IRB project number;
- A detailed description of the event, incident, experience, or outcome;
- An explanation of the basis for determining that the event, incident, experience, or outcome represents an UPIRTSO;
- A description of any changes to the protocol or other corrective actions that have been taken or are proposed in response to the UPIRTSO.

To satisfy the requirement for prompt reporting, UPIRTSOs will be reported using the following timeline:

- UPIRTSOs that are serious adverse events (SAEs) will be reported to the IRB and to the DCC/study sponsor within 7 days of the investigator becoming aware of the event.
- Any other UPIRTSO will be reported to the IRB and to the DCC/study sponsor within 7 days of the investigator becoming aware of the problem.
- All UPs should be reported to appropriate institutional officials (as required by an institution’s written reporting procedures), the supporting agency head (or designee), Food and Drug Administration (FDA), and the Office for Human Research Protections (OHRP) within 24 hours of the IRB’s receipt of the report of the problem from the investigator.

# 15. Data and Safety Monitoring Plan (DSMP)

**Data safety monitoring plan for:** Reducing Obesity Using Social Ties (ROBUST)

**Principal Investigator:** Erica Phillips, MD, MS

**Grant Application #:** 1R01DK135949-01

Overall the intervention and measurement protocols pose minimal risk to the index participants and their participating social network members. Because of this low-risk status, the data safety monitoring (DSM) plan for this trial focuses on close monitoring by the principal investigator (PI) in conjunction with a safety officer (Dr. Madeline Sterling), along with prompt reporting of excessive adverse events and any serious adverse events to the NIH and to the IRB at the Weill Cornell Medicine.

Although there are additional reports to be produced by the study coordinator as a result of this DSM plan, there are no substantive changes to the study protocol that might require review by the NIDDK. Safety reports will be sent to the study statistician, the PI, and the safety officer. The Program Manager will be responsible for assembling the data and producing these reports, as well as assuring that all parties obtain copies of these reports.

The frequency of data review for this study differs according to the type of data and is summarized in the following table:

| **Data type** | **Frequency of review** |
| --- | --- |
| Subject accrual (adherence to protocol regarding demographics, inclusion/exclusion) | Bi-annually (mid-point and end of each  study year period) |
| Adverse event rates (injuries) | As they occur |
| Intervention fidelity and adherence | Bi-annually (mid-point and end of each  study year period) |
| Stopping rules report regarding statistical power implications of dropouts and missing data | Yearly |

## Data Quality and Management

Using an Internet based system, such as REDCAP raises issues of data privacy and security. REDCap is a mature, secure web application for building and managing online surveys and databases. REDCAP has procedures and technology to restrict access to only trusted local sites. Identification of trusted sites and standard password protection techniques are employed. In addition, all data, including passwords, are encrypted using state of the art encryption software before passing across the Internet. REDCAP is fully HIPAA compliant: all users must have secure passwords of sufficient complexity that are automatically prompted to change every predefined interval. All patient privacy information is typically stored in one location (the HIPAA form), the access to which is strictly limited. The security of *REDCAP* is only part of the security of the web server (SSL encryption) and computer system being used. The servers that house *REDCAP* run SE Linux (security enhanced; the same as that used by the National Security Agency), are all password protected, are regularly backed up, and well protected. Each user entering data on the system will have security identification and specific access rights. All data will be linked to the user that entered it as well as the timestamp of data entry. After the initial data has been entered into a record, any subsequent changes of the data will be entered into a special log file and reviewed by the data management team. A complete audit trail is also built into the system: this tracks who changed what data and when. The system has multiple levels of access rights (defined as what data users are allowed to have access to) and different levels of access permission (defined as the specific use allowed to the data being accessed). Each user entering data on the system has a security identification and specific access. For example, with respect to access rights, some users would have access rights only to demographic data, but not to clinical data. With respect to access permission, some users would be given access permission only to view, but not to add, edit or delete data. Other users may have rights to add, edit, delete or view data. The data management team (Phillips, data analyst and coordinator) will establish which project members will have access and what their permission level will be.

To ensure high-quality data, in addition to range checks embedded in the RedCap forms, the research staff will review data on a weekly basis. We will investigate any data irregularities, including missing data. Monthly, they will meet with the study PI to review study data management. Any inconsistencies will lead to data queries to be resolved by the data analyst and study statistician.

In the event of an audit all requested documents will be made readily available to the regulatory body.

Protection of the participant’s privacy and confidentiality:

There is a potential risk to the participant with regards to possible violation of their privacy. We have taken steps to avoid this by making sure that only investigators on the study have access to a password protected data file. All investigators and research assistants will be required to fulfill all requirements of the institution for HIPPA and IRB training. As part of the process involved in obtaining written informed consent, all participants will be reminded that their responses are confidential and that they may refuse to participate in the project or withdraw at any time without explanation, and further, that such action will in no way affect their future interactions with their health care provider. Study data will be transmitted to the data management team for data processing using only secure methods (e.g., encryption). Prior to inclusion in any data set (internal and external), data will be stripped of all identifying information.

## Qualifications and responsibilities of the Safety Officer

The safety officer for this trial will be Madeline Sterling, MD, MPH, MS. Dr. Sterling is a practicing, board-certified general internist and a health services researcher in the Division of General Internal Medicine at Weill Cornell Medicine. She has formal training in cardiovascular clinical epidemiology, health services research (qualitative and quantitative research methods), community-engaged intervention-design, and the conduct of real-world clinical trials. She is currently PI of two community-based clinical trials, one of which involves a lifestyle intervention (NHLBI-K23HL150160; Doris Duke Clinical Scientist Award – 2022053). As such, she has an understanding of the types and severity of injuries commonly experienced as a result of lifestyle intervention. As Safety Officer, Dr. Sterling will review the reports sent by the study coordinator (at the frequency previously outlined) and will use the checklist attached to this document to determine whether there is any corrective action, trigger of an ad hoc review, or stopping rule violation that should be communicated to the study investigator, the Weill Cornell IRB, and the NIDDK.

## Measurement and reporting of subject accrual, adherence to inclusion/exclusion criteria

Review of the rate of subject accrual, adherence to inclusion/exclusion criteria will occur bi- annually (mid-point and end of each study year). This review will ensure that participants meet eligibility criteria and ethnic diversity goals outlined in the grant proposal. Based on our clinical trial milestone plan we anticipate the following accruals will have taken place at each data review.

| **Milestone #** | **Description** | **Date** |
| --- | --- | --- |
| 1 | Hire and train study staff | *Yr 1, Q2* |
| 2 | *Randomization and enrollment of 25% of participants and alters* | *Yr 1, Q4* |
| 3 | *Randomization and enrollment of 50% of participants and alters* | *Yr 2, Q2* |
| 4 | *Randomization and enrollment of 100% of participants and alters* | *Yr 2, Q4* |
| 5 | *Follow up visit completion of 100% of participants and alters; primary data collection on study participants completed; completion of close out assessment* | *Yr 3, Q2* |
| 6 | *Clean up assessment data for analysis of secondary endpoints and complete end of study summary/ data safety monitoring plan* | *Yr3, 4* |

## Measurement and reporting of adverse events

We plan to collect symptom data related to increased physical activity and adverse events as part of the routine coaching sessions at week 3, 4, 5, 7, 9, 11, 13, 15, and 20. We plan to present unblinded adverse events data to the study statistician, the PI, and the safety officer throughout this trial. There is some level of injury expected from individuals potentially increasing their physical activity although the recommended mode of physical activity will be walking and reducing sedentary time. In our previous RCT study of 405 adults enrolled in a lifestyle intervention study and followed over 12 months, 13 (16%) expected adverse events (were likely related to the study protocol. Among all AE's, 17 (21%) were related to musculoskeletal complaints. There were 7 (9%) cardiovascular events and 17 (21%) pulmonary events. 73% of the total adverse events (81) were rated as unlikely or unrelated to the intervention. ^28^

Definitions of expected adverse events for this study:

*Cardiovascular, pulmonary, hypoglycemia:* Adverse events will be defined as cardiovascular (MI, CVA, death), acute shortness of breath, or hypoglycemia precipitated by changes in dietary intake or the physical activity chosen for the study requiring hospitalization/emergency room visit or a physician office visit. Non-routine office visits for cardiac or pulmonary disease or diabetes are expected and not serious unless they lead to the above events.

*Major depressive episode or anxiety attack:* Infrequent likelihood. A participant may become depressed, anxious or severely embarrassed if friends/family/acquaintances find out that the person is participating in and/or not losing weight in the program. Patients have the option of not answering any questions they do not want to. As this is a voluntary study, a participant is free to withdraw from the study at any point if they feel uncomfortable or do not want to continue.

*Medication Side Effects* Infrequent risks. The study does not prescribe medication. Weight loss can cause a change in the dose of medication needed to treat common health conditions such as hypertension, diabetes and hypothyroidism. Participants will be required to communicate their participation in the study along with their weight loss to a primary care physician.

*Musculoskeletal*: Musculoskeletal injury due to the physical activity chosen for this study resulting in hospitalizations, emergency department visits, or treatment for fracture in any setting will be considered expected serious adverse events. Non-routine office visits for musculoskeletal injury are expected and not serious unless lead to the above events. Temporary minor incapacitation due to muscle soreness is also expected and not serious.

## Measurement and reporting of participant compliance to treatment protocol:

Session attendance will be measured as a marker of adherence, with a benchmark of attending at least 75% of the coaching/assessment sessions for both index participants (11 out of 14) and social network members (3 out of 4). 'Treatment (intervention) fidelity' will be assessed in three ways. The first is a checklist in REDCAP that the coach will complete at the end of each session. This checklist will also serve as a reminder to the coach about the active ingredients to be delivered during the session. The second method is a monthly review of a random sampling of 10% of the coaching sessions. Points will be given for each required treatment element delivered correctly and subtracted for any contamination elements. If the average fidelity falls below 90%, the coach will be retrained. The third is by each tracking the session duration in minutes. The coach will assess enactment fidelity at the beginning of each coaching session using a simple yes/no checklist. This data will be reviewed in bi-weekly team meetings (study coordinator, PI and interventionists) and reviewed by the safety officer bi-annually. If the safety officer has concerns about whether intervention fidelity has reached a level that might inhibit the ability of the study to test its primary hypotheses, she will suggest a conference call for study investigators to discuss methods for improving study adherence.

## Stopping rules

In this minimal risk lifestyle intervention trial, it is more likely that difficulty in recruiting adequate numbers of participants will require stopping the trial than that excess adverse events will occur and require stopping the trial. However, as outlined elsewhere, we will monitor injury rates in all participants and the safety officer, together with the study investigators, will alert the IRB and the NIH if a larger than reasonably expected event rate should occur in the treatment group. Other issues relating to stopping rules for this trial include:

New Information

It is exceedingly unlikely that any new information will become available during this trial that would necessitate stopping the trial.

Limits of Assumptions

It is possible that baseline differences between the groups, excessive study dropouts and/or missing data by the interim measurement time point will limit the value of data analysis of measurements at the 3-year time point. Baseline differences will be evaluated after the first measurement time point. Given the monitoring plans outlined elsewhere in this document, it is exceedingly unlikely that there will be baseline differences between groups of any magnitude to threaten the validity of the study.

While this R01 mechanism's primary aim is to determine the feasibility and acceptability of the intervention our goal is also to obtain appropriate estimates for a larger trial. Based on a sample size of 132 participants with an expected attrition of 15% we will have sufficient power (98%) to detect an 8-pound difference SD 10.4) between the intervention and control group (secondary outcome). Alert points are set at dropout rates of 30% (low alert), 40% (mid-alert), and 50% (high alert). As shown in the table below even at a high level alert we will still have moderate power (0.87) to determine our secondary outcomes.

| Dropout Rate  Alert level | 30%  low | 40%  mid | 50 %  high |
| --- | --- | --- | --- |
| Power available | 96% | 92% | 88% |

The actions taken at each level of alert are given below:

Low to mid-level alert = Conference call between study investigators to discuss approaches to minimize further losses to follow-up/dropouts.

High-level alert = Conference call between investigators to determine further alterations of study protocol to complete the study with no further losses. In the unlikely event of a 50% dropout rate occurs prior to the 6-months measurement time point, study investigators would convene on a conference call to discuss the usefulness of continuing the study.

Limits of Rules

We acknowledge that there are other situations that could occur that might warrant stopping the trial and have a section on the safety report entitled ‘Other situations that have occurred since the last safety report that warrant discussion’ to allow for communication of concerns to the study PI, statistician, and the safety officer.

# 16. References

# May AL, Freedman D, Sherry B, Blanck HM. Centers for Disease Control and Prevention (CDC); 2013

# Obesity—United States. MMWR Surveill Summ, 1999-2010; 62(Suppl 3),120-8.

1. Anekwe CV, Jarrell AR, Townsend MJ, et al. Socioeconomics of Obesity. Curr ObesRep. 2020;9(3):272–9.
2. Wang Y, Beydoun, MA. The obesity epidemic in the United States—gender, age, socioeconomic, racial/ethnic, and geographic characteristics: a systematic review and meta-regression analysis. Epidemiologic reviews. 2007;29(1), 6-28.
3. Cossrow N, Falkner B. Race/Ethnic Issues in Obesity and Obesity-Related Comorbidities. *The Journal of Clinical Endocrinology & Metabolism*. 2004; 89 (6):2590–2594, <https://doi.org/10.1210/jc.2004-0339>
4. Zhang S, de la Haye K, Ji M, An R. Applications of social network analysis to obesity: A systematic review. Obes Rev. 2018;19(7):976-988.
5. Lin J, Myers MF, Wilkinson AV, Koehly LM. Activating communal coping related to diabetes risk in mexican-heritage families. Fam Community Health. 2019;42(4):245-253.
6. de la Haye K, Bell BM, Salvy SJ. The role of maternal social networks on the outcomes of a home-based childhood obesity prevention pilot intervention. J Soc Struct. 2019;20(3):7-28.
7. Valente TW. Network interventions. Science. 2012;337(6090):49-53.
8. Hunter RF, de la Haye K, Murray JM, et al. Social network interventions for health behaviours and outcomes: A systematic review and meta-analysis. PLoS Med. 2019;16(9).
9. Frank DI, Stephens B, Lee SH. Health-promoting behaviors of African American rural women. Clin Excell Nurse Pract. 1998;2(3):159-165.
10. Chang MW, Nitzke S, Guilford E, Adair CH, Hazard DL. Motivators and barriers to healthful eating and physical activity among low-income overweight and obese mothers. J Am Diet Assoc. 2008;108(6):1023-1028.
11. Badham J, Kee F, Hunter RF. Simulating network intervention strategies: Implications for adoption of behaviour. Network Sci. 2018; 6: 265–280.
12. Elizabeth K. Ely, Stephanie M. Gruss, Elizabeth T. Luman, Edward W. Gregg, Mohammed K. Ali, Kunthea Nhim, Deborah B. Rolka, Ann L. Albright; A National Effort to Prevent Type 2 Diabetes: Participant-Level Evaluation of CDC’s National Diabetes Prevention Program. *Diabetes Care* 1 October 2017; 40 (10): 1331–1341. <https://doi.org/10.2337/dc16-2099>
13. Thornton PL, Kieffer EC, Salabarria-Pena Y, et al. Weight, diet, and physical activity-related beliefs and practices among pregnant and postpartum latino women: The role of social support. Matern Child Health J. 2006;10(1):95-104.
14. Chang MW, Baumann LC, Nitzke S, Brown RL. Predictors of fat intake behavior differ between normalweight and obese WIC mothers. Am J Health Promot. 2005;19(4):269-277.
15. Wang ML, Pbert L, Lemon SC. Influence of family, friend and coworker social support and social undermining on weight gain prevention among adults. Obesity (Silver Spring). 2014;22(9):1973-1980.
16. Spring B, Pfammatter AF, Marchese SH, et al. A factorial experiment to optimize remotely delivered behavioral treatment for obesity: Results of the opt-IN study. Obesity (Silver Spring). 2020;28(9):1652-1662.
17. Nam S, Redeker N, Whittemore R. Social networks and future direction for obesity research: A scoping review. Nurs Outlook. 2015;63(3):299-317.
18. Ajrouch KJ, Antonucci TC, Janevic MR. Social networks among blacks and whites: The interaction between race and age. J Gerontol B Psychol Sci Soc Sci. 2001;56(2):S112-8.
19. Taylor RJ, Chrs LM, Woodward AT, Brown E. Racial and ethnic differences in extended family, friendship, fictive kin and congregational informal support networks. Fam Relat. 2013;62(4):609-624.
20. Cohen S, Wills TA. Stress, social support, and the buffering hypothesis. Psychol Bull. 1985;98(2):310-357.
21. McCarty C, Lubbers M, Vacca R, Molina J. Conducting personal network research: A practical guide. New York: Guilford Press; 2019.
22. Spencer-Bonilla G, Ponce OJ, Rodriguez-Gutierrez R, Alvarez-Villalobos N, Erwin PJ, Larrea-Mantilla L, et al. A systematic review and meta-analysis of trials of social network interventions in type 2 diabetes. BMJ Open. 2017; 21: e016506
23. Wang K, Brown K, Shen SY, Tucker J. Social network-based interventions to promote condom use: a systematic review. AIDS Behav. 2011;15: 1298–1308. pmid:21811843
24. Ryan D, Heaner M. Guidelines (2013) for managing overweight and obesity in adults. Preface to the full report. Obesity (Silver Spring). 2014;22 (2): S1-3. doi: 10.1002/oby.20819. PMID: 24961822.
25. Wadden TA, Tronieri JS, Butryn ML. Lifestyle modification approaches for the treatment of obesity in adults. Am Psychol. 2020;75(2):235-251. doi: 10.1037/amp0000517. PMID: 32052997; PMCID: PMC7027681.
26. Phillips E, Charlson M, Wells M, et al. Innovative approaches to weight loss in a high risk population: The small changes and lasting effects (SCALE) trial. Obesity. 2017;25(5):833.

# Appendix A – Table 1: Schedule of Assessments


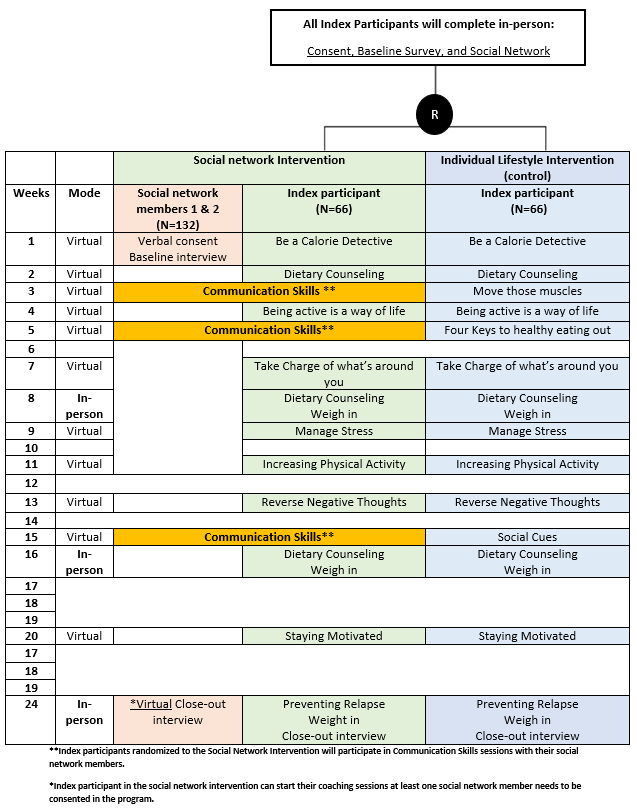


# Appendix B – Communication Skills session outline

| **SUMMARY OF ROBUST INTERVENTION SESSIONS WITH SOCIAL NETWORK MEMBERS** | | |
| --- | --- | --- |
| **Timepoint** | **Activity** | **Outcome/ Assessment** |
| Baseline – Social Network Member | - Completion of baseline survey - Program overview regarding the role of the social network member and available resources - Program overview of what the index participant will be doing - ***Assignment 1****:* Before the week 3 session instruct index participants to think about the type of the support they would like from the social network members during the program | - REDCAP survey of daily PA/SA exercise implementation during weeks 1-2 |
| Week 3 | - Coaching session will introduce elements of communal coping and review the definitions of positive affect and social undermining.   - Coach reviews ways in which social undermining can discourage positive eating and physical activity - Participant and social network member to set supportive SMART goal - ***Assignment 2:*** Participant and social network member to work on adhering to their self-selected supportive SMART goal and write down any problems with adhering | - Completion of a self-reported measure of adherence to enacting the supportive SMART goal by the alter and receipt of the SMART goal by the index participant |
| Week 5 | - Review of how to recognize social undermining and how to create positive affect in regards to engaging in positive eating & physical activity behaviors. - Coach will discuss how to use the 5 steps to problem solving in order to avoid social undermining and to encourage healthy eating out habits. | - Completion of a self-reported measure of adherence to enacting the supportive SMART goal by the alter and receipt of the SMART goal by the index participant |
| Week 15 | - Discuss usage of the positive affect and communal coping skills created to learn how to address social cues that trigger negative eating and physical activity behaviors and complete accompanying in session activity. - ***Assignment 3:*** Index participant will be asked to complete the handout on dealing with problem social cues and plan to review the responses with their social network members | - Self-reported count of the number of people that were engaged in a risk communication conversation since week 5 - Completion of a self-reported measure of adherence to enacting the supportive SMART goal by the alter and receipt of the SMART goal by the index participant |
| Week 24 | - Completion of closeout survey | - REDCAP |
