# Supplementary material for: Reducing Obesity Using Social Ties (ROBUST): Protocol for a randomized control trial of a social network lifestyle intervention
Source: PLoS One. 2025 Apr 16;20(4):e0318990. doi: 10.1371/journal.pone.0318990 (PMC12002803; doi:10.1371/journal.pone.0318990)
Supplement: S3 File — (DOC) [file pone.0318990.s003.doc]

# Data safety monitoring plan for: Reducing Obesity Using Social Ties (ROBUST)

# Principal Investigator: Erica Phillips, MD, MS

# Grant Application #: 1R01DK135949-01

Introduction

This study is a pilot randomized controlled trial comparing a social-network enhanced lifestyle intervention (ROBUST) to an individual-level lifestyle intervention (control group) in 132 Black and Hispanic adults with obesity (henceforth termed the index participant). Participants will be followed over 24 weeks and have four in-person assessments (baseline, 8 weeks, 16 weeks, and then final assessment at 24 weeks) during that period. Participants randomized to ROBUST will nominate up to two social network members (also termed alters) who will verbally consent to participation in the study supporting the index participant. The hypothesis being tested is that the social-network enhanced lifestyle intervention will be feasible, acceptable, and lead to positive change in key social network processes (ie, social undermining) that will reduce established interpersonal barriers to weight-loss. Overall the intervention and measurement protocols pose minimal risk to the index participants and their participating social network members. Because of this low-risk status, the data safety monitoring (DSM) plan for this trial focuses on close monitoring by the principal investigator (PI) in conjunction with a safety officer, along with prompt reporting of excessive adverse events and any serious adverse events to the NIH and to the IRB at the Weill Cornell Medicine.

Although there are additional reports to be produced by the study coordinator as a result of this DSM plan, there are no substantive changes to the study protocol that might require review by the NIDDK. Safety reports will be sent to the study statistician, the PI, and the safety officer. The Project Coordinator will be responsible for assembling the data and producing these reports, as well as assuring that all parties obtain copies of these reports.

The frequency of data review for this study differs according to the type of data and can be summarized in the following table:

| **Data type** | **Frequency of review** |
| --- | --- |
| Subject accrual (adherence to protocol regarding demographics, inclusion/exclusion) | Bi-annually (mid-point and end of each study year period) |
| Adverse event rates (injuries) | As they occur |
| Intervention fidelity and adherence | Bi-annually (mid-point and end of each study year period) |
| Stopping rules report regarding statistical power implications of dropouts and missing data | .Yearly |

**Data Quality and Management**

Using an Internet based system, such as REDCAP raises issues of data privacy and security. REDCap is a mature, secure web application for building and managing online surveys and databases. REDCAP has procedures and technology to restrict access to only trusted local sites. Identification of trusted sites and standard password protection techniques are employed. In addition, all data, including passwords, are encrypted using state of the art encryption software before passing across the Internet. REDCAP is fully HIPAA compliant: all users must have secure passwords of sufficient complexity that are automatically prompted to change every predefined interval. All patient privacy information is typically stored in one location (the HIPAA form), the access to which is strictly limited. The security of *REDCAP* is only part of the security of the web server (SSL encryption) and computer system being used. The servers that house *REDCAP* run SE Linux (security enhanced; the same as that used by the National Security Agency), are all password protected, are regularly backed up, and well protected. Each user entering data on the system will have security identification and specific access rights. All data will be linked to the user that entered it as well as the timestamp of data entry. After the initial data has been entered into a record, any subsequent changes of the data will be entered into a special log file and reviewed by the data management team. A complete audit trail is also built into the system: this tracks who changed what data and when.

The system has multiple levels of access rights (defined as what data users are allowed to have access to) and different levels of access permission (defined as the specific use allowed to the data being accessed). Each user entering data on the system has a security identification and specific access. For example, with respect to access rights, some users would have access rights only to demographic data, but not to clinical data. With respect to access permission, some users would be given access permission only to view, but not to add, edit or delete data. Other users may have rights to add, edit, delete or view data. The data management team (Phillips, data analyst and research assistant) will establish which project members will have access and what their permission level will be.

To ensure high-quality data, in addition to range checks embedded in the RedCap forms, the research staff will review data on a weekly basis. We will investigate any data irregularities, including missing data. Monthly, they will meet with the study PI to review study data management. Any inconsistencies will lead to data queries to be resolved by the data analyst and study statistician.

In the event of an audit all requested documents will be made readily available to the regulatory body.

**Protection of the participant’s privacy and confidentiality:**

There is a potential risk to the participant with regards to possible violation of their privacy. We have taken steps to avoid this by making sure that only investigators on the study have access to a password protected data file. All investigators and research assistants will be required to fulfill all requirements of the institution for HIPPA and IRB training. As part of the process involved in obtaining written informed consent, all participants will be reminded that their responses are confidential and that they may refuse to participate in the project or withdraw at any time without explanation, and further, that such action will in no way affect their future interactions with their health care provider. Study data will be transmitted to the data management team for data processing using only secure methods (e.g., encryption). Prior to inclusion in any data set (internal and external), data will be stripped of all identifying information.

**Qualifications and responsibilities of the Safety Officer**

The safety officer for this trial will be Madeline Sterling, MD, MPH, MS. Dr. Sterling is a practicing, board-certified general internist and a health services researcher in the Division of General Internal Medicine at Weill Cornell Medicine. She has formal training in cardiovascular clinical epidemiology, health services research (qualitative and quantitative research methods), community-engaged intervention-design, and the conduct of real-world clinical trials. She is currently PI of two community-based clinical trials, one of which involves a lifestyle intervention (NHLBI-K23HL150160; Doris Duke Clinical Scientist Award – 2022053). As such, she has an understanding of the types and severity of injuries commonly experienced as a result of lifestyle intervention. As Safety Officer, Dr. Sterling will review the reports sent by the study coordinator (at the frequency outlined above) and will use the checklist attached to this document to determine whether there is any corrective action, trigger of an ad hoc review, or stopping rule violation that should be communicated to the study investigator, the Weill Cornell IRB, and the NIDDK.

## **Measurement and reporting of subject accrual, adherence to inclusion/exclusion criteria**

Review of the rate of subject accrual, adherence to inclusion/exclusion criteria will occur bi-annually (mid-point and end of each study year). This review will ensure that participants meet eligibility criteria and ethnic diversity goals outlined in the grant proposal. Based on our clinical trial milestone plan we anticipate the following accruals will have taken place at each data review.

| **Milestone #** | **Description** | **Date** |
| --- | --- | --- |
| 1 | Hire and train study staff | *Yr 1, Q2* |
| 2 | *Randomization and enrollment of 25% of participants and alters* | *Yr 1, Q4* |
| 3 | *Randomization and enrollment of 50% of participants and alters* | *Yr 2, Q2* |
| 4 | *Randomization and enrollment of 100% of participants and alters* | *Yr 2, Q4* |
| 5 | *Follow up visit completion of 100% of participants and alters; primary data collection on study participants completed; completion of close out assessment* | *Yr 3, Q2* |
| 6 | *Clean up assessment data for analysis of secondary endpoints and complete end of study summary/ data safety monitoring plan* | *Yr3, 4* |

## **Measurement and reporting of adverse events**


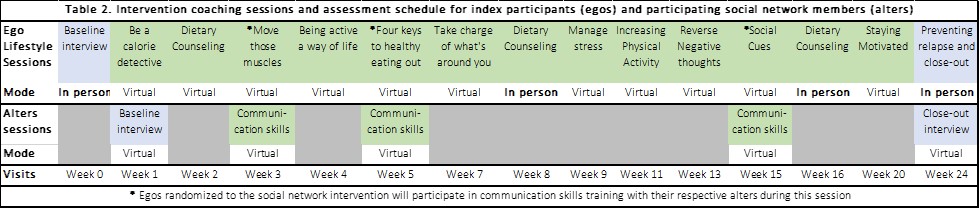
We plan to collect symptom data related to increased physical activity and adverse events as part of the routine coaching sessions following the schedule shown below.

We plan to present unblinded adverse events data to the study statistician, the PI, and the safety officer throughout this trial. The adverse event form submitted with the grant proposal has been revised to meet the goals of this data safety monitoring plan and will be used by the study staff to report injuries or other adverse events caused by the lifestyle intervention. There is some level of injury expected from individuals potentially increasing their physical activity although the recommended mode of physical activity will be walking and reducing sedentary time. In our previous RCT study of 405 adults enrolled in a lifestyle intervention study and followed over 12 months, 13 (16%) expected adverse events (were likely related to the study protocol. Among all AE's, 17 (21%) were related to musculoskeletal complaints. There were 7 (9%) cardiovascular events and 17 (21%) pulmonary events. 73% of the total adverse events (81) were rated as unlikely or unrelated to the intervention. ^1^

Definitions of expected adverse events for this study:

*Cardiovascular, pulmonary, hypoglycemia:* Adverse events will be defined as cardiovascular (MI, CVA, death), acute shortness of breath, or hypoglycemia precipitated by changes in dietary intake or the physical activity chosen for the study requiring hospitalization/emergency room visit or a physician office visit. Non-routine office visits for cardiac or pulmonary disease or diabetes are expected and not serious unless they lead to the above events.

*Major depressive episode or anxiety attack:* Infrequent likelihood. A participant may become depressed, anxious or severely embarrassed if friends/family/acquaintances find out that the person is participating in and/or not losing weight in the program. Patients have the option of not answering any questions they do not want to. As this is a voluntary study, a participant is free to withdraw from the study at any point if they feel uncomfortable or do not want to continue.

*Medication Side Effects* Infrequent risks. The study does not prescribe medication. Weight loss can cause a change in the dose of medication needed to treat common health conditions such as hypertension, diabetes and hypothyroidism. Participants will be required to communicate their participation in the study along with their weight loss to a primary care physician.

*Musculoskeletal*: Musculoskeletal injury due to the physical activity chosen for this study resulting in hospitalizations, emergency department visits, or treatment for fracture in any setting will be considered expected serious adverse events. Non-routine office visits for musculoskeletal injury are expected and not serious unless lead to the above events. Temporary minor incapacitation due to muscle soreness is also expected and not serious.

## **Measurement and reporting of participant compliance to treatment protocol**

Session attendance will be measured as a marker of adherence, with a benchmark of attending at least 75% of the coaching/assessment sessions for both index participants (11 out of 14) and social network members (3 out of 4). 'Treatment (intervention) fidelity' will be assessed in three ways. The first is a checklist that the coach will complete at the end of each session. This checklist will also serve as a reminder to the coach about the active ingredients to be delivered during the session. The second method is a monthly review of a random sampling of 10% of the coaching sessions. Points will be given for each required treatment element delivered correctly and subtracted for any contamination elements. If the average fidelity falls below 90%, the coach will be retrained. The third is by each tracking the session duration in minutes. The coach will assess enactment fidelity at the beginning of each coaching session using a simple yes/no checklist. This data will be reviewed in bi-weekly team meetings (study coordinator, PI and interventionists) and reviewed by the safety officer bi-annually. If the safety officer has concerns about whether intervention fidelity has reached a level that might inhibit the ability of the study to test its primary hypotheses, she will suggest a conference call for study investigators to discuss methods for improving study adherence.

# MP Stopping rules

In this minimal risk lifestyle intervention trial, it is more likely that difficulty in recruiting adequate numbers of participants will require stopping the trial than that excess adverse events will occur and require stopping the trial. However, as outlined elsewhere, we will monitor injury rates in all participants and the safety officer, together with the study investigators, will alert the IRB and the NIH if a larger than reasonably expected event rate should occur in the treatment group. Other issues relating to stopping rules for this trial include:

## New Information

It is exceedingly unlikely that any new information will become available during this trial that would necessitate stopping the trial.

## Limits of Assumptions

It is possible that baseline differences between the groups, excessive study dropouts and/or missing data by the interim measurement time point will limit the value of data analysis of measurements at the 3-year time point. Baseline differences will be evaluated after the first measurement time point. Given the monitoring plans outlined elsewhere in this document, it is exceedingly unlikely that there will be baseline differences between groups of any magnitude to threaten the validity of the study.

While his R01 mechanism's primary aim is to determine the feasibility and acceptability of the intervention our goal is also to obtain appropriate estimates for a larger trial. Based on a sample size of 132 participants with an expected attrition of 15% we will have sufficient power (98%) to detect an 8-pound difference SD 10.4) between the intervention and control group (secondary outcome). Alert points are set at dropout rates of 30% (low alert), 40% (mid-alert), and 50% (high alert). As shown in the table below even at a high level alert we will still have moderate power (0.87) to determine our secondary outcomes.

Dropout Rate 30% 40% 50 %

Alert level low mid high

Power available 96% 92% 88%

The actions taken at each level of alert are given below:

Low to mid-level alert = Conference call between study investigators to discuss approaches to minimize further losses to follow-up/dropouts.

High-level alert = Conference call between investigators to determine further alterations of study protocol to complete the study with no further losses. In the unlikely event of a 50% dropout rate occurs prior to the 6-months measurement time point, study investigators would convene on a conference call to discuss the usefulness of continuing the study.

### Definitions

**Withdrawn** refers to a subject’s decision to discontinue participation in the study. This decision can occur for various reasons, such as personal preferences, health concerns, or logistical issues.

**Dropout** will refer to participants who, after consenting and randomization, fail to proceed to the onboarding appointment (week 0) or complete the baseline questionnaire. Drop-out participants will need to be replaced.

**Lost to follow-up** is any participant who has completed the baseline questionnaire and week 0 enrollment but fails to respond to future contact after any coaching sessions.

### Limits of Rules

We acknowledge that there are other situations that could occur that might warrant stopping the trial and have a section on the safety report entitled ‘Other situations that have occurred since the last safety report that warrant discussion’ to allow for communication of concerns to the study PI, statistician, and the safety officer.

Table 1a. ENROLLMENT BY MONTH OF STUDY: INDEX PARTICIPANTS

**Date: _________________________**
 

| **Month** | **# Expected to randomize** | **# Signed consent** | **# Randomized** | # Withdrawn | #Drop out | #Lost to follow-up |
| --- | --- | --- | --- | --- | --- | --- |
| APRIL |  |  |  |  |  |  |
| MAY |  |  |  |  |  |  |
| JUNE |  |  |  |  |  |  |
| JULY |  |  |  |  |  |  |
| AUGUST |  |  |  |  |  |  |
| SEPTEMBER |  |  |  |  |  |  |
| OCTOBER |  |  |  |  |  |  |
| NOVEMBER |  |  |  |  |  |  |

SCREENING, ENROLLMENT, AND EXCLUSION SUMMARY

|  | **TOTAL** |
| --- | --- |
| # EMR screened |  |
| # Excluded after completing EMR screening |  |
| # Passed the EMR screen and invite to participate |  |
| # Scheduled Q&A about study |  |
| # Attended study Q&A |  |
| # Consented |  |
| #Eligible for randomization |  |
| # Randomized |  |
| #Completed baseline interview |  |
| **Reasons for Exclusion** |  |
| Under 18 years old |  |
| Does not have at least one social network member that will participate |  |
| Active enrollment in a weight-loss program, use of weight-loss medications or planning weight-loss surgery |  |
| Advanced medical illness, dementia, hospitalization, injury, or pregnancy that inhibits regular physical activity |  |
| Contraindication to exercise based on Physical Activity Readiness Questionnaire or lack of clearance from a health care provider. |  |

Table 1b. ENROLLMENT BY MONTH OF STUDY: SOCIAL NETWORK PARTICIPANTS

**Date: _________________________**
 

| **Month** | **# Electronically consent** | **# Withdrawn** | **#Dropped from study** |
| --- | --- | --- | --- |
|  |  |  |  |
|  |  |  |  |
|  |  |  |  |
|  |  |  |  |
|  |  |  |  |
|  |  |  |  |
|  |  |  |  |
|  |  |  |  |

SCREENING, ENROLLMENT, AND EXCLUSION SUMMARY

|  | **TOTAL** |
| --- | --- |
| # Referred by participants |  |
| #Contacted |  |
| # Electronically consented |  |
| #Completed baseline interview |  |
| **Reasons for Exclusion** |  |
| Under 18 years old |  |
| Does not speak English or Spanish |  |
| Does not live in the United States |  |

Table 2a. RACE/ETHNIC CHARACTERISTICS OF INDEX PARTICIPANTS

**Date: _________________________**
 

| **Total Enrollment Report: Number of Subjects Enrolled to Date by Ethnicity and Race** | | | | |
| --- | --- | --- | --- | --- |
| **Ethnic Category** | **Sex/Gender** | | | |
|  | **Females** | **Males** | **Unknown** | **Total** |
| Hispanic or Latino |  |  |  |  |
| Not Hispanic or Latino |  |  |  |  |
| Unknown |  |  |  |  |
| *Ethnic Category: Total of All Subjects* |  |  |  |  |
| **Racial Categories** |  | | | |
| American Indian/Alaska Native |  |  |  |  |
| Asian |  |  |  |  |
| Native Hawaiian or Pacific Islander |  |  |  |  |
| Black or African American |  |  |  |  |
| White |  |  |  |  |
| More than one race |  |  |  |  |
| Unknown or unreported |  |  |  |  |
| ***Racial Categories: Total All Subjects*** |  |  |  |  |
|  | | | | |
| **HISPANIC ENROLLMENT REPORT: Number of Hispanics or Latinos Enrolled to Date** | | | | |
| **Racial Categories** |  |  |  |  |
| American Indian/Alaska Native |  |  |  |  |
| Asian |  |  |  |  |
| Native Hawaiian or Pacific Islander |  |  |  |  |
| Black or African American |  |  |  |  |
| White |  |  |  |  |
| More than one race |  |  |  |  |
| Unknown or unreported |  |  |  |  |
| ***Racial Categories: Total All Subjects*** |  |  |  |  |

 

Table 2b. RACE/ETHNIC CHARACTERISTICS OF SOCIAL NETWORK PARTICIPANTS

**Date: _________________________**
 

| **Total Enrollment Report: Number of Subjects Enrolled to Date by Ethnicity and Race** | | | | |
| --- | --- | --- | --- | --- |
| **Ethnic Category** | **Sex/Gender** | | | |
|  | **Females** | **Males** | **Unknown** | **Total** |
| Hispanic or Latino |  |  |  |  |
| Not Hispanic or Latino |  |  |  |  |
| Unknown |  |  |  |  |
| *Ethnic Category: Total of All Subjects* |  |  |  |  |
| **Racial Categories** |  | | | |
| American Indian/Alaska Native |  |  |  |  |
| Asian |  |  |  |  |
| Native Hawaiian or Pacific Islander |  |  |  |  |
| Black or African American |  |  |  |  |
| White |  |  |  |  |
| More than one race |  |  |  |  |
| Unknown or unreported |  |  |  |  |
| ***Racial Categories: Total All Subjects*** |  |  |  |  |
|  | | | | |
| **HISPANIC ENROLLMENT REPORT: Number of Hispanics or Latinos Enrolled to Date** | | | | |
| **Racial Categories** |  |  |  |  |
| American Indian/Alaska Native |  |  |  |  |
| Asian |  |  |  |  |
| Native Hawaiian or Pacific Islander |  |  |  |  |
| Black or African American |  |  |  |  |
| White |  |  |  |  |
| More than one race |  |  |  |  |
| Unknown or unreported |  |  |  |  |
| ***Racial Categories: Total All Subjects*** |  |  |  |  |

 

Table 3a. BASELINE CHARACTERISTICS AND DEMOGRAPHICS OF INDEX PARTICIPANTS

**Date: _________________________**
 

| **Characteristics** | **Treatment**  **N%** | **Control**  **N%** | **Total**  **N%** |
| --- | --- | --- | --- |
| **Sex** |  |  |  |
| - Male |  |  |  |
| - Female |  |  |  |
| **Ethnicity** |  |  |  |
| Hispanic or Latino |  |  |  |
| Not Hispanic or Latino |  |  |  |
| Unknown |  |  |  |
| Race |  |  |  |
| - American Indian/Alaska Native |  |  |  |
| - Asian |  |  |  |
| - Native Hawaiian or Other  Pacific Islander |  |  |  |
| - Black or African American |  |  |  |
| - White |  |  |  |
| - Hispanic or Latino |  |  |  |
| - More than one race |  |  |  |
| - Unknown or not reported |  |  |  |
| **Age** |  |  |  |
| - Mean |  |  |  |
| - Median |  |  |  |
| - Minimum |  |  |  |
| - Maximum |  |  |  |
| **Clinical Features** |  |  |  |
| Mean weight |  |  |  |
| BMI mean |  |  |  |
| BMI median |  |  |  |
| Body fat % measured by BIA |  |  |  |

Table 3b. BASELINE CHARACTERISTICS AND DEMOGRAPHICS OF SOCIAL NETWORK PARTICIPANTS

**Date: _________________________**
 

| **Characteristics** | **Treatment**  **N%** | **Control**  **N%** | **Total**  **N%** |
| --- | --- | --- | --- |
| **Sex** |  |  |  |
| - Male |  |  |  |
| - Female |  |  |  |
| **Ethnicity** |  |  |  |
| Hispanic or Latino |  |  |  |
| Not Hispanic or Latino |  |  |  |
| Unknown |  |  |  |
| Race |  |  |  |
| - American Indian/Alaska Native |  |  |  |
| - Asian |  |  |  |
| - Native Hawaiian or Other  Pacific Islander |  |  |  |
| - Black or African American |  |  |  |
| - White |  |  |  |
| - Hispanic or Latino |  |  |  |
| - More than one race |  |  |  |
| - Unknown or not reported |  |  |  |
| **Age** |  |  |  |
| - Mean |  |  |  |
| - Median |  |  |  |
| - Minimum |  |  |  |
| - Maximum |  |  |  |
| **Clinical Features** |  |  |  |
| Mean weight |  |  |  |
| BMI mean |  |  |  |
| BMI median |  |  |  |

Table 4. TREATMENT DURATION FOR ALL SUBJECTS

**Date: _________________________**
 

| **TIME IN STUDY** | **N** | **%** | **TOTAL** |
| --- | --- | --- | --- |
| Less than 4 weeks |  |  |  |
| 4-7 weeks |  |  |  |
| 8-11 weeks |  |  |  |
| 12-16 weeks |  |  |  |
| 17-23 weeks |  |  |  |
| Completed study |  |  |  |

Table 5. TREATMENT DURATION FOR SUBJECTS WHO DISCONTINUED THERAPY

**Date: _________________________**
 

| **Time in Study** | **N** | **%** | **Total** |
| --- | --- | --- | --- |
| Less than 4 weeks |  |  |  |
| 4-7 weeks |  |  |  |
| 8-11 weeks |  |  |  |
| 12-16 weeks |  |  |  |
| 17-23 weeks |  |  |  |
| Completed study |  |  |  |

Table 6. ADVERSE EVENTS

**Date: _________________________**

| **Subject** | **Adverse Event** | **Onset Date** | **Ending Date** | ***Severity** | ***Inter Related** | ***Action** | ***Outcome** | Comments |
| --- | --- | --- | --- | --- | --- | --- | --- | --- |
|  |  |  |  |  |  |  |  |  |
|  |  |  |  |  |  |  |  |  |
|  |  |  |  |  |  |  |  |  |
|  |  |  |  |  |  |  |  |  |

**CODES:

Severity: Physical Activity/ Dietary Relatedness:**

1 = Mild 0 = Definitely unrelated

2 = Moderate 1 = Unlikely

3 = Severe 2 = Possibly Related

4 = Life threatening** 3 = Probably Related

4 = Definitely Related

| **Action (taken):** | **Outcome:** |
| --- | --- |
| 0 = None | 1 = Resolved |
| 1 = Exercise modification | 2 = Recovered with minor changes to daily activities |
| 2 = Minor counteractive medical treatment | 3 = Recovered with major changes to daily activities |
| (specify under comments) | 4 = Condition still present and under treatment |
| 3 = Major medical intervention | 5 = Condition continues to worsen |
| (specify under comments) | 6 = Patient died** |
| 4 = Hospitalization** |  |
| 5 = Other (specify under comments) |  |
|  |  |
|  |  |

****Event is serious and explained in detail on SAE form**

Table 7. SERIOUS ADVERSE EVENTS

**Date: _________________________**
 

| **Subject** | **Age** | **Treatment Date** | **Event** | **Onset Date** | ***Relationship** | **Description of Actions and Outcome(e.g., hospitalization concomitant meds, study, status, etc.)** |
| --- | --- | --- | --- | --- | --- | --- |
|  |  |  |  |  |  |  |
|  |  |  |  |  |  |  |
|  |  |  |  |  |  |  |
|  |  |  |  |  |  |  |
|  |  |  |  |  |  |  |

***RELATIONSHIP**
0 = Definitely Unrelated

1 = Unlikely

2 = Possibly Related

3 = Probably Related

4 = Definitely Related

Table 8. DEATHS

**Date: _________________________**
 

| **Patient ID#** | **DOB** | **Date Enrolled** | **Treatment Duration** | **Cause of Death** | **Date of Death** |
| --- | --- | --- | --- | --- | --- |
|  |  |  |  |  |  |
|  |  |  |  |  |  |
|  |  |  |  |  |  |
|  |  |  |  |  |  |
|  |  |  |  |  |  |
|  |  |  |  |  |  |
|  |  |  |  |  |  |

Table 9. FREQUENCY OF SPECIFIC SYMPTOMS (Index participants only)

**Date: _________________________**
 

| **Symptoms** | **N%** |
| --- | --- |
| Joint pain |  |
| Soft tissue pain (more than soreness from exercise) |  |
| Pain or Heaviness in Chest with physical activity |  |
| Increased heart rate (palpitations) |  |
| Shortness of breath with physical activity |  |
| Feeling dizzy and weak |  |
| Lightheadedness during physical activity or at rest |  |
| Excessive sweating |  |
| Sudden confusion |  |
| Total |  |

**Checklist for safety officer to complete after reviewing the safety report:**

Please reply in the right column

| 1. Were participants accrued at a rate of 8 per month during the recruitment period? |  |
| --- | --- |
| 1. Do all recruited subjects meet eligibility criteria? |  |
| 1. Are the dropouts above 15%? |  |
| 1. Is the overall musculoskeletal (joint and soft tissue pain) injury rate for this quarter above 21% in either group? |  |
| 1. Is the overall rate of cardiovascular (pain or heaviness in the chest) or pulmonary (shortness of breath) symptoms complaint rate for this quarter above 21% in either group? |  |
| 1. What is the % adherence (out of total possible) for this period of engagement in coaching sessions? |  |
| 1. Are there any other situations that have occurred in the trial since the last safety report that warrant triggering a study investigator conference call or contact with the WCM IRB or NIDDK? (If yes, please explain in the space below this checklist) |  |
| 1. Based on any of the replies above, is there any need to communicate with the WCM IRB and/or the NIDDK regarding data monitoring or safety of participants? |  |
| 1. Based on your review of the safety report, is there need for discussion between study investigators regarding data monitoring and/or participant safety? |  |
| 1. Do you currently have any conflicts of interest that may influence your ability to objectively evaluate the data provided by this study? |  |
